# Functional and mechanistic studies of a phytogenic formulation, Shrimp Best, in growth performance and vibriosis in whiteleg shrimp

**DOI:** 10.1038/s41598-024-62436-x

**Published:** 2024-05-21

**Authors:** Yi-San Lee, Khotibul Umam, Tien-Fen Kuo, Yu-Liang Yang, Ching-Shan Feng, Wen-Chin Yang

**Affiliations:** 1 Agricultural Biotechnology Research Center, Academia Sinica, Taipei, Taiwan; 2 Institute of Biotechnology, National Taiwan University, Taipei, Taiwan; 3 Graduate Institute of Biotechnology, National Chung Hsing University, Taichung, Taiwan; 4 Molecular and Biological Agricultural Sciences, Taiwan International Graduate Program, Academia Sinica, Taipei, Taiwan; 5 National Chung Hsing University, Taichung, Taiwan; 6 Faculty of Life Science and Technology, Biotechnology, Sumbawa University of Technology, Sumbawa, Indonesia; 7 Graduate Institute of Integrated Medicine, China Medical University, Taichung, Taiwan

**Keywords:** Gut microbiota, Growth performance, Phytogenic, *Vibrio*, Antimicrobial metabolites, Fatty acids, Animal biotechnology, Marine biology

## Abstract

Climate change and disease threaten shrimp farming. Here, we studied the beneficial properties of a phytogenic formulation, Shrimp Best (SB), in whiteleg shrimp. Functional studies showed that SB dose-dependently increased shrimp body weight and decreased feed conversion ratio. We found that SB protected against *Vibrio parahaemolyticus* as evidenced by survival rate, bacterial load, and hepatopancreatic pathology in shrimp. Finally, we explored the likely mechanism by which SB affects growth performance and vibriosis in shrimp. The 16S rRNA sequencing data showed that SB increased 6 probiotic genera and decreased 6 genera of pathogenic bacteria in shrimp. Among these, SB increased the proportion of *Lactobacillus johnsonii* and decreased that of *V. parahaemolyticus* in shrimp guts. To dissect the relationship among SB, *Lactobacillus* and *Vibrio*, we investigated the in vitro regulation of *Lactobacillus* and *Vibrio* by SB. SB at ≥ 0.25 μg/mL promoted *L. johnsonii* growth. Additionally, *L. johnsonii* and its supernatant could inhibit *V. parahaemolyticus*. Furthermore, SB could up-regulate five anti-*Vibrio* metabolites of *L. johnsonii*, which caused bacterial membrane destruction. In parallel, we identified 3 fatty acids as active compounds from SB. Overall, this work demonstrated that SB improved growth performance and vibriosis protection in shrimp via the regulation of gut microbiota.

## Introduction

The World Food and Agriculture Organization estimates that global shrimp production exceeds 7 million tons, with a market value of US$133.43 billion, of which about 54% comes from artificial farming. The annual market for shrimp feed and its supplements is $5 billion and $100 million, respectively^1,2^. Whiteleg shrimp (*Litopenaeus vannamei*) and tiger shrimp (*Penaeus monodon*) are the two major species of shrimp used in commercial production in aquaculture^3^. Due to the environment and pathogens, high-density shrimp farming is prone to infectious disease outbreaks which cause economic losses^3^. To reduce infectious diseases, specific pathogen-free post-larvae, a sanitary environment, and biosecurity measurements are needed^4^. Prophylaxis and therapy for shrimp diseases are important in shrimp farming. Antibiotics are commonly used to prevent and treat bacterial infections in shrimp. However, they can leave drug residues, or result in drug-resistant pathogens and an imbalance of gut bacteria, leading to food safety and public health issues^5^. More importantly, prolonged use of antibiotics damages the animal’s gut system, including changing the gut microbiome and depressing the immune system and gut health^5^. Furthermore, animals do not readily return to normal growth performance after drug withdrawal^5^.

Reduction of antibiotics can only be achieved when there are other effective approaches to prevent shrimp disease and increase shrimp health. Several studies have revealed that gut microbiota play a critical role in strengthening host health, growth performance^6,7^, immunity^8^ and protection against pathogens^6^. Both beneficial and harmful bacteria are present in the gut microbiota of hosts. Out of these bacteria, *Lactobacillus* is a genus that is popularly known as a probiotic which has various health benefits. *Lactobacillus* is gram-positive, rod-shaped, facultative anaerobic, and non-spore-forming^9^. In addition, *Megasphaera*^10^, *Bifidobacterium*^11^, *Prevotella*^10^, *Ruminococcus*^12^ and *Collinsella*^13^ have also been reported to be probiotics that positively impact host growth in shrimp and other animals^11,14^. In contrast, harmful gut bacteria have adverse effects on the host growth performance in shrimp by triggering malfunctions and diseases. Such bacteria include *Vibrio*, *Photobacterium*, *Pseudoalteromonas*, *Planctomicrobium*, *Tenacibaculum* and *Corynebacterium_1*^15^. The balance of probiotics and pathogens in the gut is achieved through multiple mechanisms such as competitive exclusion, production of antimicrobial substances, nutrient competition, quorum sensing, and immune modulation^16^. For instance, lactic acid (LA), acetic acid (AA), propionic acid (PA), butyric acid (BA), and 3-hydroxypropionic acid (3-HPA) derivative have been well documented to be produced by *Lactobacillus* and other gut bacteria^17^. Moreover, these metabolites have been shown to inhibit the growth of pathogenic bacteria via direct bactericidal action on pathogens and indirect action on host cells^18^.

Medicinal plants are emerging as an attractive alternative to antibiotics. Over 300,000 species of flowering plants worldwide constitute an extraordinary source of bacteriostats and bactericidals in aquatic as well as food animals^19^. Phytogenics are defined as a single chemical or a mixture of chemicals of plant origin. They can replace and reduce the use of antibiotics, and increase the survival rate and growth performance of aquatic organisms^3^. The advantages of phytogenics are that they contain multifunctional phytochemicals, have no drug residue problems, and have low levels of development of drug resistance. Their disadvantages are their high chemical complexity, difficulty in identifying active ingredients, unclear mechanisms, time labor requirements, and batch efficacy vairation^3^. Effective phytogenics for infectious diseases are in high demand^3,20,21^. Among them, bitter melon (*Momordica charantia*) has been reported to protect shrimp against viruses^22^. Of note, Spanish needles (*Bidens pilosa*) is listed as an edible *Asteraceae* herb. This plant has been demonstrated to modulate microbiota in chicken gut as well as humans via increasing probiotics and decreasing pathogenic bacteria^20^. Mechanistic studies showed that Spanish needles increased probiotics (*e.g.*, *Lactobacillus* and *Bifidobacterium*) and reduced pathogenic bacteria in chicken gut^20^. However, the probiotic-promoting compounds in Spanish needles have not been identified so far. We and other groups have identified over 300 compounds from *B. pilosa*^23,24^. Of note, 12 long fatty acids were found to be present at high levels in this plant, including behenic acid, 2-butoxyethyl linoleate, ethyl linolenic acid, methyl linolenate, linolenic acid, linoleic acid, capric acid, elaidic acid, myristic acid, lauric acid, palmitic acid, and palmitoleic acid^24^. One publication stated that linolenic acid and linoleic acid at 10 to 40 μg/mL inhibited the growth of *Lactobacillus* species^25^. Conversely, another publication showed that linolenic acid increased the proportion of *Lactobacillus* and *Bifidobacterium* and decreased that of *Enterococcus* and *E. coli *in vitro using rat fecal fermentation system^26^. Furthermore, oleic acid but not palmitoleic acid was reported to promote the growth of *Lactobacillus*^27^. Nevertheless, little is known about the mechanism by which long-chain fatty acids regulate gut microbiota.

In this study, we evaluated the function of one phytogenic formulation, Shrimp Best (SB), containing Spanish needles and bitter melon, shrimp growth performance and *Vibrio* infection. We then investigated the likely mechanism through which SB regulates gut microbiota. Lastly, we identified the active compounds in SB that regulate probiotics.

## Materials and methods

### Chemicals, reagents, *bacteria*, and bacterial media

LA, AA, PA, BA, 3-HPA, sodium chloride (NaCl), linolenic acid, linoleic acid, stearic acid, palmitic acid, HCl, methanol, ampicillin (Amp), and chloramphenicol (Chl) were purchased from Sigma-Aldrich (Munich, Germany). ^13^C6-3NPH·HCl (purity ≥ 98%), N-(3-dimethylaminopropyl)-N′-ethylcarbodiimide (EDC) and pyridine were purchased from Cayman (Ann Arbor, MI). Shrimp Best (SB), comprising a standardized extract of Spanish needles and bitter melon at an equal ratio, was manufactured by Eubiotics (Taiwan) using good manufacturing practice guidelines. This extract is a fine, light olive green powder with a relatively uniform granularity. Individual particles are irregular in shape with a particle size of 100–200 microns. High-pressure liquid chromatography was used for quality control. Tryptone Soy broth (TSB) or agar (TSA), de Man, Rogosa, and Sharpe (MRS) medium, thiosulfate-citrate-bile salts-sucrose (TCBS) media, and agar were purchased from Becton Dickinson (Sparks, MD). Plate count agar was purchased from Sigma-Aldrich. *L. johnsonii* (ATCC 33,200) and *V. parahaemolyticus* (ATCC 17,802) were cultured with shaking under aerobic and anaerobic conditions, respectively, at 37 °C for 16 h. Amp (30 µg/mL) and Chl (25 µg/mL) were used in this study unless indicated otherwise.

### Feed preparation

The remainder of the diet was made up of standard diet (No. 1 shrimp feed, Shye-Yih Feeding, Taiwan) composed of 35% crude protein, 2.8% lipid and 3% fiber. For the control group, the feed was pelleted into 1.4 mm. For the other groups, the crushed diet was mixed with SB and sterile water (0.5 L/kg), pelleted and dried. First, a safe dosage of SB was determined by testing SB as 0–10% of the total feed administered in five groups of shrimp. SB at 0–5% showed no obvious adverse effect on shrimp based on body weight (CTR and SB, Fig. S1). However, SB at 10% failed to increase body weight (10% SB, Fig. S1), implying a likely adverse effect on shrimp. In the interest of cost-effectiveness and shrimp performance, SB at 0%, 0.04%, 0.2% and 1% were selected as the dosage range for subsequent studies. Four groups of specific pathogen-free whiteleg shrimp, 27 shrimp per group, were then assigned as described in Fig. S2A. They were fed with standard diet (control group) or a diet containing 0.04% 0.2% and 1% of SB (treatment groups) for 28 days.

### Animal

#### Study

For growth performance study in the laboratory, specific pathogen-free whiteleg shrimp at 79 days of age were purchased from the Department of Aquaculture, National Taiwan Ocean University, Taiwan. They were raised and acclimatized in a maintenance tank (2 × 1 × 0.5 m^3^) for 7 days and fed with the test diets until they reached a size of 3.28 ± 0.30 g. A total of 108 shrimp were assigned into 4 groups, one control and three treatment groups (SB 0.04%, SB 0.2% and SB 1%). Each group had 3 replicates, 9 shrimp per replicate. Three replicates of each group were performed. Shrimp were housed at a stocking density of 9 individuals per aquarium (60 × 25 × 30 cm^3^) and fed to satiation with an initial ratio of 5% biomass, equally divided into 3 rations per day (administered at 9:00, 15:00 and 21:00) for 28 days. The diet was adjusted daily based on consumption. The aquaria were equipped with aerators and top filters. Thirty percent of the water was replaced in each aquarium every three days. Dissolved oxygen (DO, 9.6 ± 1.21 mg/L), temperature (28.29 ± 0.98 °C), pH (8.19 ± 0.2), salinity (15.5 ± 0.99 g/L), total ammonia nitrogen (0.20 ± 0.13 mg/L) and nitrite (0.49 ± 0.14 mg/L) were recorded. Body weight (BW), body length (BL), food consumption, and feed conversion rate (FCR) of each group were measured weekly. BL was measured from the base of the eye stalk of the shrimp to the tail fan using ImageJ software.

For field trial, shrimp at 9 days post larvae (PL-9, ~ 16 days of age) were purchased from the hatchery of Charoen Pokphand, Thailand, and acclimatized in indoor maintenance tanks. PL-9 shrimp were housed at a density of 5000 individuals per tank (2 × 1 × 0.5 m^3^) and fed with copepods for 2 weeks. At 30 days of age, the shrimp were transferred to three outdoor earthen ponds for an additional 135-day grow-out as outlined in Table 1. Three groups, a control (CTR) and two treatment groups receiving SB at doses of 0.2% (SB 0.2%) and 1% (SB 1%), each group housed in a separate pond, were fed daily. They were fed to satiation with an initial ratio of 5% biomass, equally divided into 4 rations per day (administered at 5:00, 9:00, 16:00 and 21:00), for the last 2 months, followed by a ratio of 2% biomass until harvest. The ponds were equipped with aerators. Ten to twenty percent of water was exchanged daily in each pond. Ambient temperature (30.16 ± 0.86 °C), DO (9.57 ± 0.23 mg/L), pH (8.14 ± 0.25), salinity (20.20 ± 0.14 g/L), total ammonia nitrogen (0.20 ± 0.13 mg/L), and nitrite (0.47 ± 0.02 mg/L) were recorded. Shrimp number, stocking density, BW, BL, food consumption, biomass, and FCR were measured. During the grow-out period, a sampling net (80 × 80 cm^2^) with the diet was placed vertically into the water, left for 20 min, and pulled up vertically to catch shrimp. Body weight and BL of the shrimp were measured.

**Table 1:** Growth performance of shrimp in a field trial over 135 days.

Parameters	CTR	SB 0.2%	SB 1%
Area of the pond (m^2^)	2194	3263	4323
Initial shrimp number	150,000	200,000	300,000
Stocking density (shrimp/m^2^)	68.37	61.29	69.4
Initial BW (g)	0.38 ± 0.03	0.37 ± 0.05	0.37 ± 0.02
Initial BL (mm)	28.58 ± 3.34	28.02 ± 3.38	28.14 ± 2.27
Final BW (g)	22.38 ± 0.63	25.51 ± 1.44^a^	26.29 ± 0.86^b^
Final BL (mm)	118.59 ± 2.57	136.54 ± 7.93^a^	142.66 ± 4.68^b^
BW gain (g)	22 ± 0.62	25.14 ± 1.42^a^	25.92 ± 0.85^a^
Food intake (g)	37.04 ± 1.04	39.15 ± 2.17^a^	39.50 ± 1.27^a^
FCR	1.72 ± 0.0	1.56 ± 0.06^a^	1.52 ± 0.02^a^
Days of culture	135	135	135
Harvest (kg)	3357	5102	7887

Results are presented as Mean ± SD. One-way ANOVA was used to compare the difference of mean among CTR, SB 0.2% and SB 1%. The letters a and b indicate statistical significance (*P* < 0.05) between the CTR and SB-treated groups, SB 0.2% and SB 1%, respectively.

For shrimp challenge study with *V. parahaemolyticus* in the laboratory, 100 whiteleg shrimp, aged 176 days, with an average BW of 20 g, were randomly divided into 5 groups, 20 shrimp in each group. Non-infected (NIC) and infected groups (IC) were fed with the standard diet whilst the other three groups, SB 0.04%, SB 0.2% and SB 1%, were fed with the diet containing SB at the dosage of 0.04%, 0.2% and 1%, respectively. On day 28, shrimp in the IC group and SB treatment groups received an intra-muscular injection of *V. parahaemolyticus* (5 × 10^4^ CFU/g BW). The shrimp in each group were measured daily for survival, bacterial load in different organs, and histochemical pathology of hepatopancreas. All animals were handled based on the guidelines of the Institutional Animal Care and Use committee (IACUC) Academia Sinica, Taiwan, and the experimental methods were carried out in accordance with relevant guidelines and regulations of the ARRIVE guidelines.

### DNA purification and metagenomic analysis of gut microbiota in shrimp

**Figure 1:**
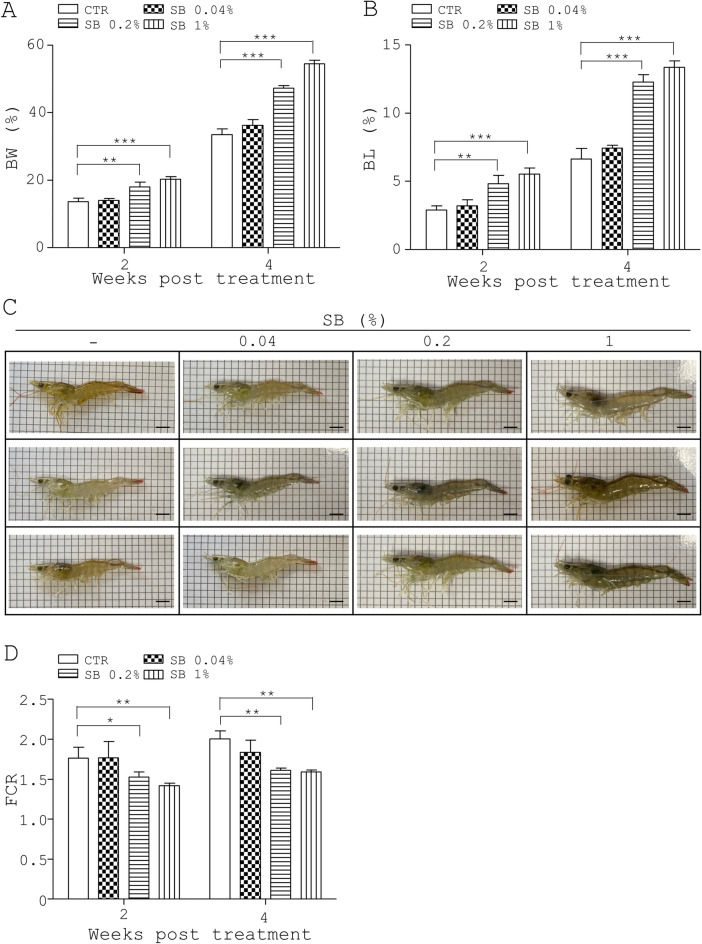
Beneficial effects of SB on growth performance in shrimp. (**A**–**D**) Four groups of 86-day-old shrimp, 12 animals a group, were fed with a standard diet (CTR) or the diet containing SB at 0.04%, 0.2%, and 1% for 4 weeks. Their BW (**A**), BL (**B**), photograph (**C**) and food conversion ratio (FCR, **D**) were measured. Data in triplicate or more are presented as mean ± standard deviation. One-way ANOVA test was used for statistical analysis of differences between groups and *P* (*) < 0.05, *P* (**) < 0.01, and *P* (***) < 0.001 are considered statistically significant. The number of animals is indicated in the parenthesis.

Four groups of shrimp, shown in Fig. 1, were fed with standard diet (control group) or diets containing 0.04% 0.2% and 1% of SB (treatment groups) for 28 days. To collect the digestive tract of the shrimp groups, shrimp were fasted for 24 h to reduce the feed remaining in the digestive tract. Nine shrimp per group were randomly selected to collect the whole digestive tract of shrimp and three intestinal digesta were pooled with a total of three replicates a group. The bacterial DNA of each group was extracted using PrestoTM Stool DNA Extraction Kit Quick Protocol (Geneaia Biotech, Taiwan). DNA concentration and quality were determined using NanoDrop ND-1000 (Thermo-Fisher Scientific, USA). NGS and three replicates of each group were used to analyze the intestinal flora of shrimp by PCR.

For 16S rRNA NGS, bacterial DNA was amplified using PCR with 16S rRNA primers (5′-TCGTCGGCAGCGTCAGATGTGTATAAGAGACA-GCCTACGGGNGGCWGCAG-3′ and 5′-GTCTCGTGGGCTCGGAGATGTGTATAA-GAGACAGGACTACHVGGGTATCTAATCC-3′) and sequenced (NovaSeq sequencing 6000, Illumina, San Diego, CA). The 16S rRNA sequences were trimmed using Chimera Check and analyzed using the RDP pipeline Classifier with the GreenGenes database (gg_13_8; default). Next, the R program (v.3.3.1) was used to classify the operational taxonomic unit (OTU) of bacteria, which was presented by the rarefaction curve and the composition of the bacterial genus.

### Semi-quantitative PCR

PCR was performed to detect *L. johnsonii 16S rRNA* gene and *V. parahaemolyticus irgB* gene using different groups of gut metagenomic DNA together with their primer pairs as listed in Table 2. Briefly, the reaction contained 12.5 μl 2X SuperRed MasterMix, loading dye (Biotools, Taiwan), 1 μl of forward and reverse primers (10 mM, Blirt, Poland) and bacterial DNA. The PCR condition for *L. johnsonii 16S rRNA gene* was, denaturation (94 °C × 5 min), amplification [(94 °C × 30 s), annealing (54 °C × 30 s) and extension (72 °C × 30 s) for the indicated cycles)] and final extension (72 °C × 5 min). The same PCR condition was used for the *V. parahaemolyticus irgB* gene except that the annealing temperature was changed to 52 °C. The PCR products underwent gel electrophoresis (2% gel wt/vol and 1% safe dye) and were visualized using the high-resolution fluorescent biomedical imaging system v.8.21.18 (Analytik, CA, USA). The signal of the above genes in gut microbiomes was quantified using VisionWorks software. PCR was conducted on 3 biological replicates in each metagenomic sample.

**Table 2:** Specific primers used to quantify the genes present in the shrimp intestinal bacteria.

Gene	Primer name	Forward/reverse sequence	Amplicon length	GeneBank accession number
*16S rRNA*	LjF	5′-ATAACAACACTAGACGCATG-3′	134	MW368810.1
	LjR	5′-AGTCTCTCAACTCGGCTATG-3′		
*irgB*	VpF	5′-CGATACACACCACGATCCAG-3′	369	CP003972.1
	VPR	5′-GTGATGTTTCTCATACCCTTG-3′		

### Isolation, screening, and identification of shrimp gut *bacteria*

The guts of three shrimp were pooled, homogenized, and incubated in TSB medium and MRS medium, containing 3% sodium chloride, to select *Vibrio* and *Lactobacillus*, respectively, under facultative anaerobic conditions at 37 °C for one day. After streaking, a single colony of *Vibrio* and *Lactobacillus* species was isolated on TCBS plates containing 3% sodium chloride and MRS plates containing 3% sodium chloride, respectively.

### Shrimp gut *bacteria* identification (MALDI-TOF)

Sixteen to 48 colonies of *Vibrio* and *Lactobacillus* species were amplified in TSB and MRS medium, respectively. After extensive washing, the bacteria were centrifuged, and pelleted. After treatment with 70% formic acid using Vortex, bacteria were pulled down by centrifugation at 13,000 rpm for 2 min. The bacteria were mixed with 70% formic acid and 100% acetonitrile in an equal ratio, followed by 3 freeze/thaw cycles using liquid nitrogen and 1 cycle of ultrasound sonication at 4 °C for 20 min. The bacterial homogenates were dissolved in matrix solution (10 mg α-cyano-4-hydroxycinnamic acid in 1 mL standard solution: 50% acetonitrile and 2.5% trifluoroacetic acid) and subjected to MALDI-TOF-MS (Bruker, Billerica, MA) as described^28^. Briefly, protein extract (1 μL) was placed on the MTP 384 target plate (Bruker). After air drying, 1 μL of matrix solution was dropped onto the target sample. The targets were analyzed by Autoflex Speed MALDI-TOF/TOF mass spectrometry (Bruker) in the linear positive mode in the range of m/z 2000–20,000 (Detector Gain 8.1 × 2819 V) and the laser frequency was 500 Hz. The laser pulses of each target were 1500 laser shots from three 500 laser shots per position and five spectra were collected from five targets. The standards of calibration were Protein Calibration Standard for Mass Spectrometry (Bruker) and the total protein extracted from *Escherichia coli* DH5α, and then the DH5α was used as a positive control of identification by Biotyper 3.1 (Bruker) with scores higher than 2.3 against the DH5α main spectrum profile in the in-house Biotyper database.

### Minimum activatory concentration test, minimum inhibitory concentration test and half maximal inhibitory concentration

The minimum activatory concentration (MAC) and minimum inhibitory concentration (MIC) of SB for bacteria were measured in six replicates as published^29^. For MAC tests, *L. johnsonii* (1 × 10^4^ CFU/mL) was incubated with SB (0.25–4 µg/mL), Amp (30 µg/mL), 0.1% peptone, and 0.1% methanol (NC) at 37 °C for 11 h. For MIC tests, *V. parahaemolyticus* (2 × 10^4^ CFU/mL) was incubated with SB (100–1600 µg/mL), Amp (30 µg/mL), and 0.1% methanol at 37 °C for 6 h. Bacterial growth (%) was calculated as a ratio of optical density at 600 nm (OD_600_) of the treatment group to that of the NC group multiplied by 100. Alternatively, the half maximal inhibitory concentration (IC_50_) value of each antimicrobial metabolite against both pathogens was determined using OD_600_.

### Disc diffusion assays and agar well diffusion assays

To test antagonism between *Lactobacillus* and *Vibrio*, *V. parahaemolyticus* (2 × 10^5^ CFU) and *Vibrio* spp. (2 × 10^5^ CFU) were grown on TSA at 37 °C for 12 h, respectively. MRS agar, a paper disc containing water (150 µl) and Chl (0.125 µg), and agar discs containing *L. reuteri* (2 × 10^5^ CFU) and *L. johnsonii* (2 × 10^5^ CFU) , were placed on the top of TSA at 37 °C for additional 12 h. Likewise, *L. reuteri* (2 × 10^5^ CFU) and *L. johnsonii* (2 × 10^5^ CFU) were grown on MRS agar plates for 12 h. TSB (150 µl), a paper disc containing water (150 µl) and Chl (0.125 µg), and agar disc containing *V. parahaemolyticus* (2 × 10^5^ CFU) and *Vibrio* spp. (2 × 10^5^ CFU) , were placed on the top of MRS plates at 37 °C for an additional 12 h. The diameter of the inhibition zone was photographed and measured using ImageJ software. Similar to disc diffusion assays, agar well diffusion assays on *Vibrio* species grown by TSA was used to measure the anti-bacterial activity of *Lactobacillus* supernatants and metabolites, which were held in agar wells.

### Propidium iodide staining and transmission *electron* microscopy analysis

*V. parahaemolyticus* (5 × 10^3^ CFU/mL) was treated with each antimicrobial metabolite at different dosages to determine its IC_50_ value against the pathogen for 30 min. Alternatively, the bacteria were treated with Amp (30 μg/mL) and a mixture of antimicrobial metabolites, which equaled the composition of the metabolites in cell-free supernatants of SB-treated *L. johnsonii* for 7 h, at the indicated time. The bacteria were divided into 3 aliquots. One aliquot was stained with propidium iodide (PI; 20 μg/mL), and visualized using a fluorescent microscope. Another aliquot was stained with PI, and analyzed with a LSR II flow cytometer (BD Biosciences, Franklin Lakes, NJ) and FlowJo software. The other aliquot was analyzed using TEM (FEI Company, Hillsboro, OR).

### Histochemical staining of shrimp hepatopancreases

Three animals per group were sacrificed 72 h after infection. Their hepatopancreases were removed, fixed with Davison’s solution (34.7% water, 11.1% acetic acid, 32% ethanol, and 22.2% formaldehyde, 10% phosphate buffered saline) for 48 h. The tissues were dehydrated as follows: 70% alcohol (50 min), 80% alcohol (50 min), 90% alcohol (50 min), 95% alcohol (50 min), 95% alcohol for 1 h, 100% alcohol for 50 min, 100% alcohol for 50 min, and 100% alcohol (1 h). After alcohol removal with 100% xylene (2 h), the tissues were mounted in paraffin, sectioned into 5-μm slides, and stained with hematoxylin and eosin. The slides were photographed under an upright microscope.

### Bacterial counting in shrimp organs

Three shrimp from each group in the challenge study were sacrificed 3 days post *Vibrio* infection. Their stomach, gut, muscle, and hepatopancreases were removed, weighed and homogenized. The homogenates of the stomachs, guts, muscles, and hepatopancreata were diluted to 2 × 10^4^, 2 × 10^4^, 5 × 10^3^, and 1 × 10^4^ times, respectively, with 0.85% NaCl and grown on TCBS plates overnight. The average number of bacteria from each tissue (in triplicate plating), was calculated.

### Quantification of antimicrobial metabolites in shrimp gut digesta using LC–MS/MS

Gut digesta from shrimp fed with the standard diet and the diet containing SB for 4 weeks were collected. Their aqueous extracts (40 μl) were incubated with ^13^C6-3NPH·HCl solution to conjugate with antimicrobial metabolites in the supernatants as published^30^. Briefly, the conjugation reaction of ^13^C6-3NPH·HCl with metabolites at room temperature in the presence of EDC and pyridine followed a nucleophilic addition–elimination reaction mechanism, converting the metabolites to their 3-NPH derivatives. The reaction mixtures were analyzed using the Acquity UPLC- Xevo TQ-XS triple quadrupole mass spectrometer (Waters, Millford, MA) with an ESI source in negative mode.

### Identification and quantification of active compounds in SB extract and its fractions using GC-GC/MS

Derivatization of fatty acid methyl esters (FAME) was conducted as published^31^. Briefly, the dried SB extract and fractions were dissolved in 5% HCl in methanol and incubated at 85 °C for 2.5 h. After cooling to room temperature, water and hexane at a ratio of 1:2 was used to extract the FAME. The top organic layer was harvested, dried, and analyzed using GC-GC/MS (Pegasus 4D GC × GC-TOFMS fitted with a 30 m × 0.25 mm × 0.25 μm Rtx-5MS column, Restek, USA). Matches for fatty acids in the above samples were confirmed manually and by searching the National Institute of Standards and Technology (NIST) database using the NIST Mass Spectral Search Program. The fatty acids in the SB extract and fractions were quantified based on normalization to the internal standards as described^31^.

### Statistical analysis

All the data are expressed as mean ± standard deviation. One-way ANOVA was used for statistical analysis among groups. Log-rank (Mantel-Cox) test was used to compare shrimp survival between control and treatment groups. *P* values less than 0.05 (*), less than 0.01 (**), and less than 0.001 (***) were considered statistically significant.

## Results

### Beneficial effects of SB on growth performance in whiteleg shrimp

First, we examined the effect of SB at 0%, 0.04%, 0.2% and 1% on the growth performance of whiteleg shrimp for 4 weeks in a laboratory setting. Growth performance metrics, including BW, BL, food consumption, and FCR of each group, were measured at the indicated times (Table S1). The control group and three treatment groups (SB 0.04%, SB 0.2% and SB 1%) had average initial BW of 3.26 g, 3.27 g, 3.29 g and 3.31 g, respectively (Table S1). No significant difference was observed among the four groups. The control group and three treatment groups (SB 0.04%, SB 0.2% and SB 1%) showed increased average BW (13.68%, 14.05%, 17.99% and 20.34%, respectively) at 2 weeks post treatment (2 weeks, Fig. 1A). There was statistical significance between the control and two treatment groups (SB 0.2% and SB 1%) (2 weeks, Fig. 1A). The control group and three treatment groups (SB 0.04%, SB 0.2% and SB 1%) also showed increased average BW (33.5%, 36.25%, 47.23%, and 54.43%, respectively) at 4 weeks post treatment (4 weeks, Fig. 1A). There was statistical significance between the control and two treatment groups (SB 0.2% and SB 1%) (4 weeks, Fig. 1A).

The control group and three treatment groups (SB 0.04%, SB 0.2%, and SB 1%) had average initial BL of 65.75 mm, 65.85 mm, 65.68 mm, and 66.11 mm, respectively (Table S1). No significant difference was observed among the four groups. The control group and three treatment groups (SB 0.04%, SB 0.2% and SB 1%) showed increased average BL (2.90%, 13.20%, 4.82% and 5.53% respectively) 2 weeks post treatment (2 weeks, Fig. 1B). There was statistical significance between the control and two treatment groups (SB 0.2% and SB 1%) (2 weeks, Fig. 1B). The control group and three treatment groups (SB 0.04%, SB 0.2% and SB 1%) showed increased average BL (6.63%, 7.45%, 12.30%, and 13.37%, respectively) at 4 weeks post treatment (4 weeks, Fig. 1B). There was statistical significance between the control and two treatment groups (SB 0.2% and SB 1%) (4 weeks, Fig. 1B). Representative photographs of each shrimp group are shown (4 weeks, Fig. 1C).

Furthermore, the control group and three treatment groups (SB 0.04%, SB 0.2% and SB 1%) had an average FCR of 1.76, 1.77, 1.53, and 1.42, respectively, 2 weeks post treatment (2 weeks, Fig. 1D). There was statistical significance between the control and two treatment groups (SB 0.2% and SB 1%) (2 weeks, Fig. 1D). Likewise, the control group and three treatment groups (SB 0.04%, SB 0.2% and SB 1%) had an average FCR of 2, 1.84, 1.61, and 1.59, respectively, 4 weeks post treatment (4 weeks, Fig. 1D). Statistical significance was found between the control and two treatment groups (SB 0.2% and SB 1%) (4 weeks, Fig. 1D). Overall, the above data indicated that SB dose-dependently promoted the growth performance of shrimp and that the minimum effective dose was 0.2%.

Since water quality is important for shrimp farming, next, water quality metrics were monitored for 28 days in a laboratory setting (Table S2). The average water temperature of the four shrimp groups was 28.11–28.55 °C. Their average salinity was 15.17–15.96% and their average pH value was 7.96–8.56. In addition, the average dissolved oxygen was 9.36–9.67 mg/mL and the average nitrite was 0.35–0.66 mg/mL. The average ammonia nitrogen of the three shrimp groups was 0.12–0.29 mg/mL.

Finally, we assessed the effect of SB on shrimp growth over 135 days in a field trial (Table 1). The data showed that the initial BW of shrimp in the control and two treatment groups (SB 0.2% and SB 1%) were 0.38 g, 0.37 g and 0.37 g, respectively (Table 1). No significant difference was observed in the initial BW among the four groups (Table 1). The control and treatment groups (SB 0.2% and SB 1%) showed increased average BW to 22.38 g, 25.51 g and 26.29 g, respectively, 135 days post treatment (Table 1). Statistical significance between the control and two treatment groups (SB 0.2% and SB 1%) was found in the final BW (Table 1).

The initial BL of shrimp in the control and two treatment groups (SB 0.2% and SB 1%) were 28.58 mm, 28.02 mm, and 28.14 mm, respectively (Table 1). No significant difference was observed among the three groups (Table 1). Similarly, the control and treatment groups (SB 0.2% and SB 1%) had average final BL of 118.59 mm, 136.54 mm, and 142.66 mm, respectively (Table 1). Statistical significance was found between the control and two treatment groups (SB 0.2% and SB 1%) (Table 1). Overall, the above data indicated that SB dose-dependently promoted the growth performance of shrimp in farm trials.

Water quality metrics in this trial were monitored for 135 days (Table S3). The average water temperature of the water housing the four shrimp groups was 29–30 °C. The average salinity was 20% and the average pH value was 7.89–8.39. In addition, the average dissolved oxygen was 9.32–9.77 mg/mL and the average nitrite was 0. 53–0.8 mg/mL. The average ammonia nitrogen for the three shrimp groups was 0.45–0.49 mg/mL.

### SB protects against *V. parahaemolyticus* infection in shrimp

**Figure 2:**
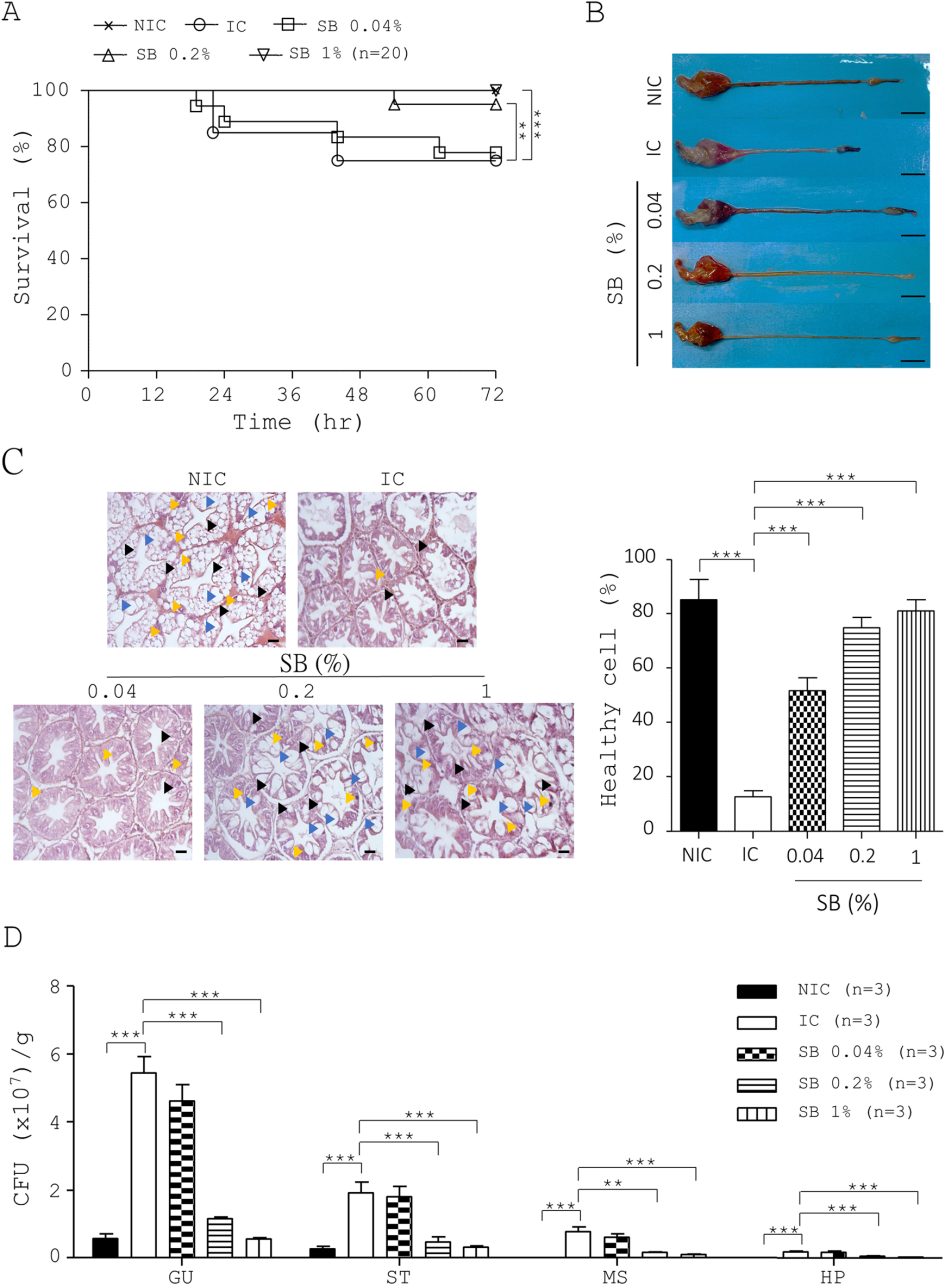
Protective effects of SB on survival rate, hepatopancreatic structure and bacterial counts in different organs of shrimp following *V. parahaemolyticus* infection. (**A**–**D**) Five groups of shrimp were fed with control diets and diets containing 0.04%, 0.2% and 1% SB (Groups 3–5), followed by infection with *V. parahaemolyticus*. The survival rate (**A**), digestive tract (**B**), hepatopancreatic structure (left, **C**), percentage of healthy hepatopancreatic cells (right, **C**), and bacterial load (**D**) in the gut (GU), stomach (ST), muscle (MS), and hepatopancreas (HP) of the un-medicated non-infected controls (NIC), un-medicated infected controls (IC) and SB-treated shrimp groups (0.04%, 0.2% and 1% SB) were measured. Blue arrowhead, yellow arrowhead, and black arrowhead indicate B, F and R cells of hepatopancreas.

Next, we tested the effect of SB on shrimp in protecting against vibriosis (Fig. S2B) based on survival, bacterial counts, and hepatopancreatic pathology. As expected, the NIC group had 100% survival (NIC, Fig. 2A). However, the IC group had a survival rate of 75% (IC, Fig. 2A). In contrast, SB dose-dependently increased this survival rate (SB, Fig. 2A). Furthermore, we examined hepatopancreatic pathology in the 5 shrimp groups. First, gross examination data showed that the IC shrimp had a yellowish to whitish hepatopancreas compared to the black hepatopancreas in the NIC shrimp (top, IC vs. NIC, Fig. 2B). However, SB treatment improved the hepatopancreatic pathology based on its color (top, SB, Fig. 2B). Next, the IC group had a shortened gut compared to the NIC group. In sharp contrast, SB had a longer gut than IC group (IC vs. SB, Fig. 2B). Accordingly, hepatopancreatic morphology data indicated that SB treatment improved the hepatopancreatic pathology (middle, SB, Fig. 2B). In parallel, microscopic examination showed that shrimp without *V. parahaemolyticus* challenge had a normal architecture of hepatopancreas with a high number of B, F, and R cells (top, NIC, Fig. 2C). In contrast, *V. parahaemolyticus* profoundly damaged the hepatopancreatic structure and reduced the number of B, F, and R cells in the shrimp hepatopancreas (top, IC, Fig. 2C). However, SB treatment dose-dependently reduced hepatopancreatic lesions and restored the number of B, F, and R cells in the shrimp hepatopancreas (bottom, SB, Fig. 2C). We also determined the percentage of healthy cells of shrimp hepatopancreas (right, Fig. 2C). Finally, we determined the bacterial counts in different organs of five shrimp groups (SB, Fig. 2D). First, few bacteria in the hepatopancreas of the NIC shrimp grew on TCBS plates (NIC, Fig. 2D). As expected, the hepatopancreas of IC shrimp showed the most bacteria on TCBS plates (IC, Fig. 2D). However, SB dose-dependently reduced the bacteria in the hepatopancreas of the infected shrimp (SB, Fig. 2D). Likewise, SB dose-dependently reduced bacterial counts in the muscle (SB, Fig. 2D), stomach (SB, Fig. 2D), and gut (SB, Fig. 2D) of the infected shrimp. Taken together, SB effectively rescued shrimp from vibriosis.

### SB increases probiotics and reduces pathogenic *bacteria* in shrimp gut

**Figure 3:**
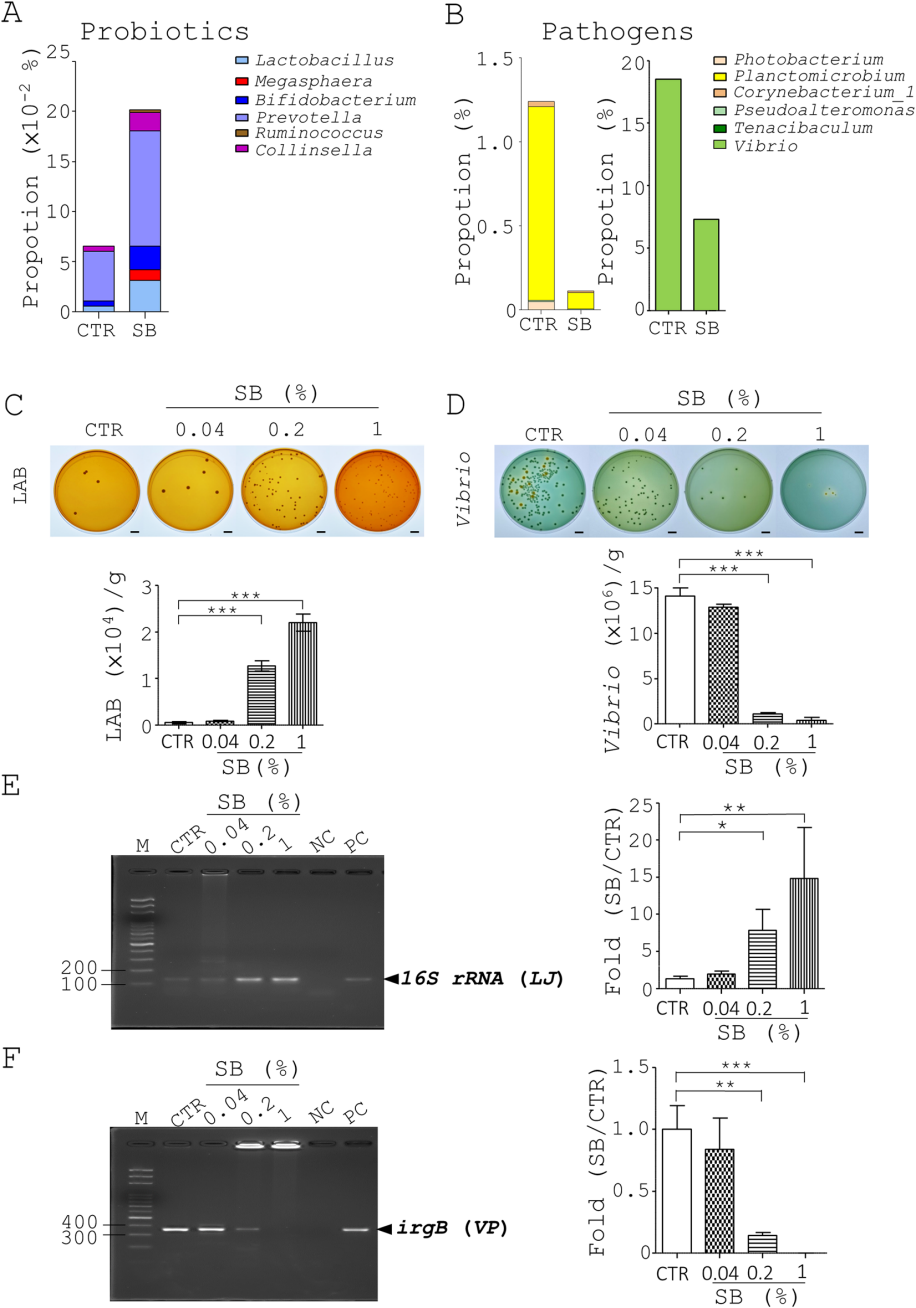
SB increases 6 probiotic genera and decreases 6 genera of pathogenic bacteria in shrimp guts. (**A**–**B**) The whole gut of 114-day-old shrimp, 9 animals a group, from 4 groups (Fig. 1) were collected to obtain gut bacteria DNA, followed by bacterial 16S rRNA NGS analysis. The proportion of probiotics (**A**) and pathogens (**B**) of the gut microbiota were analyzed at the genus level. (**C**–**D**) The same gut bacteria from Fig. 3A and B were plated on MRS plates and TCBS plates for bacterial counting (top). The count of *Lactobacillus* (LAB, **C**) and *Vibrio* species (**D**) in each group was re-plotted into histograms (bottom). (**E**–**F**) Primers specific for *L. johnsonii* (**E**) and *Vibrio parahaemolyticus* (**F**) were used to quantify the abundance of both species in the shrimp gut microbiota (Fig. 3A and B) using PCR (left). The fold change of both species was re-plotted into histograms (right). DNA marker (M) and amplicon of gut bacterial DNA of shrimp fed with SB (0.04%, 0.2%, and 1%), water (NC) and DNA of *L. johnsonii* or *Vibrio parahaemolyticus* (PC). Data from 3 repeats are presented as the mean ± SD. One-way ANOVA test was used for statistical analysis of differences between groups and *P* (*) < 0.05, *P* (**) < 0.01, and *P* (***) < 0.001 are considered statistically significant.

To explore the mechanism through which SB affects growth performance and resistance to *Vibrio* in whiteleg shrimp, the gut microbiota of shrimp were analyzed using 16S rRNA next-generation sequencing (NGS) analysis. The number of sequences, operational taxonomic units (OTUs), and diversity indices in the gut digesta of control shrimp and shrimp fed with SB (1%) are summarized in Table S4. Rarefaction curves showed that the number of sequences from 2 shrimp groups were enough to reveal the major OTUs (Fig. S3A). The gut microbiota of control shrimp (CTR) aged 126 days, had higher diversity than SB-fed shrimp as evidenced by Shannon and Chao1 diversity indices (Table S4). Detailed changes at the phylum, class, order, family, and genus levels under SB treatment are shown in Table S5 and Fig. S2B–E. Nine genera of probiotics and 10 genera of pathogens were identified from shrimp guts (Genus, Table S5). Among them, SB increased 6 beneficial bacterial genera, *Lactobacillus*, *Megasphaera*, *Bifidobacterium*, *Prevotella*, *Ruminococcus* and *Collinsella* (Fig. 3A). In contrast, SB decreased 6 harmful bacterial genera, *Vibrio*, *Photobacterium*, *Pseudoalteromonas*, *Planctomicrobium*, *Tenacibaculum* and *Corynebacterium_1* (Fig. 3B). Next, we used selective medium plates to analyze the change in the number of *Lactobacillus* species and *Vibrio* species in the intestinal digesta of 4 shrimp groups 28 days post treatment. We found that SB significantly increased the proportion of *Lactobacillus* species in the shrimp digesta in a dose-dependent fashion using the lactic acid bacteria screening medium, MRS (Fig. 3C). However, SB significantly decreased the proportion of *Vibrio* species in the shrimp digesta in a dose-dependent manner using the *Vibrio* screening medium, TCBS (Fig. 3D).

Furthermore, we used MALDI-TOF MS to identify *Lactobacillus* species as well as *Vibrio* species in the intestinal digesta of control whiteleg shrimp (Fig. S4). MS profiles of bacterial proteins were searched against an in-house database using Biotyper 3.1 as published^28^. The data showed the dominant presence of *L. johnsonii* which was more prevalent than *L. reuteri* in the gut digesta of control shrimp (Table 3). We also discovered the dominant presence of *V. parahaemolyticus*, which as more prevalent than one unidentified *Vibrio* spp. in the shrimp digesta (Table 3). Hereafter, *L. johnsonii*, *L. reuteri*, *V. parahaemolyticus*, and/or *Vibrio* spp. were used in the study.

**Table 3:** Identification of gut bacteria of shrimp using MALDI-TOF MS analysis.

Bacteria	Colony number	Score*	Note
*Enterococcus faecalis*	8	> 2	
*L. johnsonii*	22	> 2	
*Lactobacillus* spp.	4	1.8–2	Identified only at genus level
*L. reuteri*	10	> 2	
*Enterococcus avium*	2	> 2	
*Enterococcus* spp.	2	1.8–2	Identified only at genus level
*V. parahaemolyticus*	13	> 2	
*Vibrio* spp.	3	1.8–2	Identified only at genus level

Different numbers of bacterial colonies from shrimp guts were isolated for identification. *A log score between 0 and 3 is calculated by the Biotyper algorithm. The log score < 2.0 indicates genus identification whilst the log score ≥ 2 indicates species identification.

Finally, we confirmed the presence of *L. johnsoni* and *V. parahaemolyticus* in the gut digesta of the four shrimp groups from Fig. 1 using semi-quantitative polymerase chain reaction (PCR) with a primer pair of *16S rRNA* of and *irgB*, respectively. We demonstrated that SB significantly increased the proportion of *Lactobacillus* species in the shrimp digesta in a dose-dependent manner (Figs. 3E and S8A). On the contrary, SB significantly decreased the proportion of *Vibrio* species in the shrimp digesta in a dose-dependent manner using the *Vibrio* screening medium, TCBS (Figs. 3F and S8B).

Collectively, the overall data demonstrated that SB regulated the intestinal flora of shrimp mainly through augmenting probiotics and reducing pathogens.

### SB inhibits the growth of *V. parahaemolyticus *via an increase of *L. johnsonii* growth and its antimicrobial metabolites

**Figure 4:**
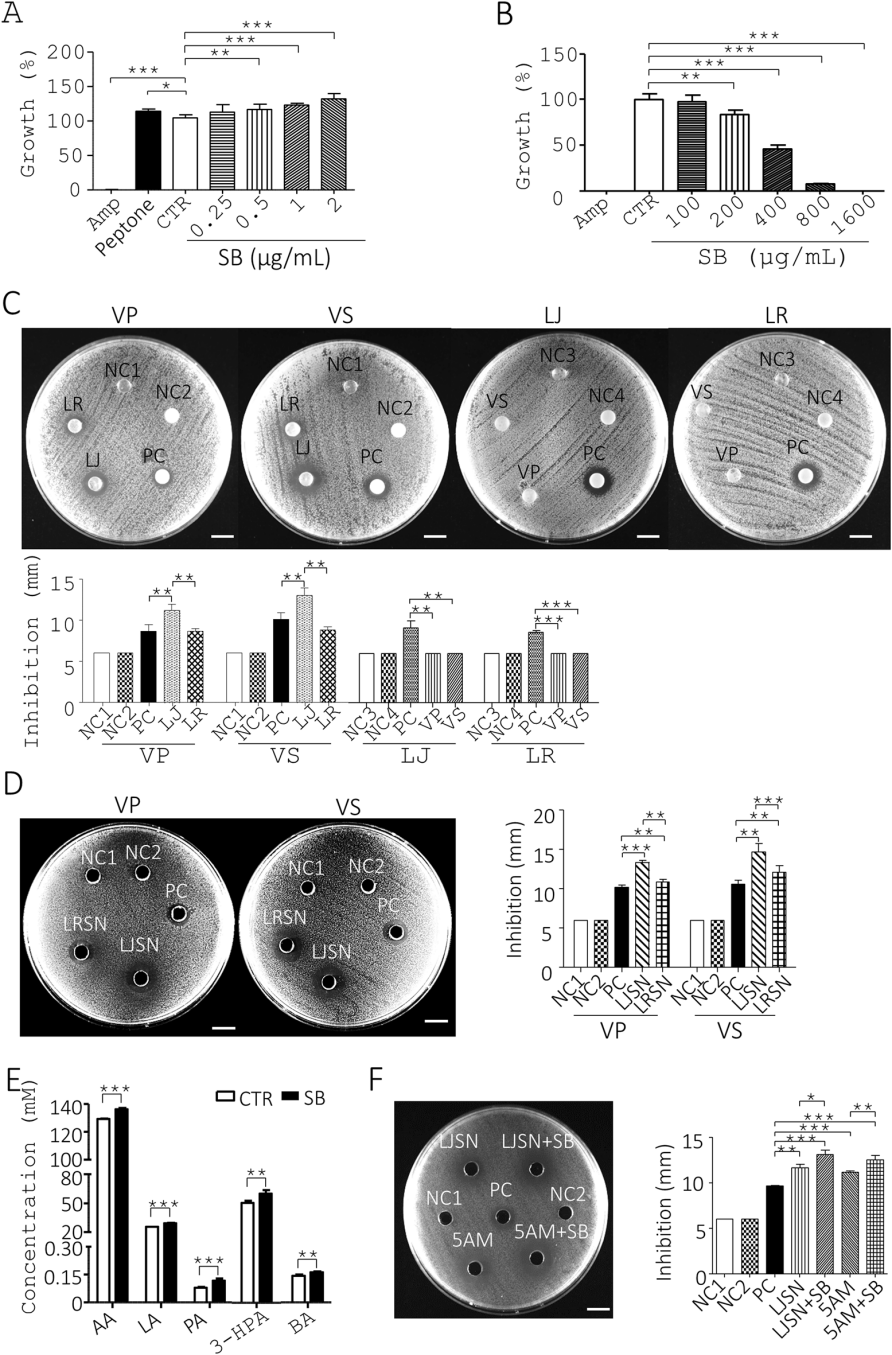
SB suppresses *Vibrio* growth via up-regulation of *Lactobacillus* growth and its antimicrobial metabolites. (**A**) *L. johnsonii* (LJ) was cultured in MRS medium containing Amp, peptone, and SB at the indicated dosages at 37 °C under anaerobic conditions for 11 h. Bacterial growth rate (%) is shown. (**B**) The same procedure as (**A**) was performed except that *V. parahaemolyticus* (VP) was grown in TSB under aerobic conditions for 6 h. (**C**) Antagonism of *L. reuteri* (LR) and LJ toward pathogens. VP (1st column, top) and *Vibrio spp*. (VS) (2nd column, top) were spread on TSA and incubated with MRS agar (NC1), a paper disc containing 150 µl water (NC2) and 0.125 µg Chl (PC). Conversely, LJ (3rd column, top) and LR (4th column, top) were spread on MRS agar plates and incubated with TSA (NC3), and a paper disc containing 150 µl water (NC4) and 0.125 µg Chl (PC). Their inhibition zones are shown. (**D**) Antagonism of the metabolites of LJ toward pathogens. VP (1st column, left) and VS (2nd column, left) were spread on TSA and incubated with 150 µl MRS medium (NC1), 150 µl water (NC2), and a hole containing 0.125 µg Chl (PC), and 150 µl supernatant of LJ (LJSN) and LR (LRSN). Their inhibition zones are shown. (**E**) SB up-regulated 5 antimicrobial metabolites of LJ as characterized in Fig. S5. (**F**) Antagonism of the mixture of 5 metabolites of SB-treated LJ toward VP. VP was spread on TSA and incubated with 150 µl MRS medium (NC1), 150 µl water (NC2), 0.125 µg Chl 150 µl (PC), the supernatant of LJ (LJSN) and SB-treated LJ (LJSN + SB), and the mixture of five metabolites (5AM) present in the supernatant of LJ (5AM) and SB-treated LJ (5AM + SB). Their inhibition zones are shown. Data from 3 repeats are presented as the mean ± SD. One-way ANOVA test was used for statistical analysis of differences between groups and *P* (*) < 0.05, *P* (**) < 0.01, and *P* (***) < 0.001 are considered statistically significant. Scale bar = 10 mm.

To tease out the mechanism through which SB promoted probiotics and inhibited pathogenic bacteria, we first tested the in vitro effects of SB on growth of *L. johnsonii*. The MAC experiments indicated that a negative control, 30 µg/mL Amp, completely inhibited the growth of *L. johnsonii* in MRS medium (Amp, Fig. 4A). In sharp contrast, a positive control, 0.1% peptone, increased the growth of *L. johnsonii* (Peptone, Fig. 4A). Of note, SB at 0.5 µg/mL and more significantly promoted the growth of *L. johnsonii* in a dose-dependent manner (SB, Fig. 4A). Next, we assessed the in vitro effects of SB on growth of *V. parahaemolyticus*. The MIC experiments showed that a positive control, 30 µg/mL Amp, completely inhibited the growth of *V. parahaemolyticus* in TSB medium (Amp, Fig. 4B). However, SB at 200 µg/mL and more significantly inhibited the growth of *V. parahaemolyticus* in a dose-dependent manner (SB, Fig. 4B). The data suggested that SB directly promoted growth of probiotics, which antagonized pathogens.

Next, we performed antagonistic tests between two *Lactobacillus* species, *L. johnsonii* (LJ) and *L. reuteri* (LR), and two *Vibrio* species, *V. parahaemolyticus* (VP) and *Vibrio* spp. (VS) using disc diffusion assays. As expected, two negative controls, MRS agar and water, could not inhibit *V. parahaemolyticus* as characterized by lack of inhibition zone (NC1 and NC2, VP (1st column), Fig. 4C). In contrast, a positive control (PC), a disc containing 0.125 µg Chl, inhibited the growth of *V. parahaemolyticus* as shown by inhibition zones (PC, VP (1st column), Fig. 4C). Likewise, a disc containing *L. johnsonii* and *L. reuteri* significantly inhibited *V. parahaemolyticus* as inhibition zones (LJ and LR, VP (1st column), Fig. 4C). We noticed that *L. johnsonii* exhibited slightly better inhibition of *V. parahaemolyticus* than *L. reuteri* and the positive control, a disc containing 0.125 µg Chl (LJ, LR and PC, VP (1st column), Fig. 4C). In parallel, we conducted antagonistic tests between two *Lactobacillus* species and *Vibrio* spp. Using disc diffusion assays. *L. johnsonii* exhibited slightly better inhibition of *Vibrio* spp. Than *L. reuteri* and the positive control, a disc containing 0.125 µg Chl (LJ, LR, and PC, VS (2nd column), Fig. 4C).

Furthermore, disc diffusion assays were applied to test the action of *V. parahaemolyticus* and *Vibrio* spp. On two *Lactobacillus* species. As expected, two negative controls, TSA and water vehicle, failed to inhibit *L. johnsonii* as characterized by the lack of an inhibition zone (NC3 and NC4, LJ (3rd column), Fig. 4C). In contrast, a positive control (PC), a disc containing 0.125 µg Chl, inhibited growth of *L. johnsonii* as shown by inhibition zones (PC, LJ (3rd column), Fig. 4C). Likewise, discs containing *V. parahaemolyticus* and *Vibrio* spp. failed to inhibit the growth of *L. johnsonii* based on inhibition zones (VP and VS, LJ (3rd column), Fig. 4C). In parallel, we found that the positive control, a disc containing 0.125 µg Chl, could inhibit the growth of *L. reuteri* (PC, LR (4th column), Fig. 4C). However, no antagonism of two *Vibrio* species, *V. parahaemolyticus* and *Vibrio* spp., and two negative controls TSA and water, toward *L. reuteri* was seen (VP, VS, NC3 and NC4, LR (4th column), Fig. 4C). The overall data revealed antagonism of *Lactobacillus* species toward *Vibrio* species and not vice versa.

**Figure 5:**
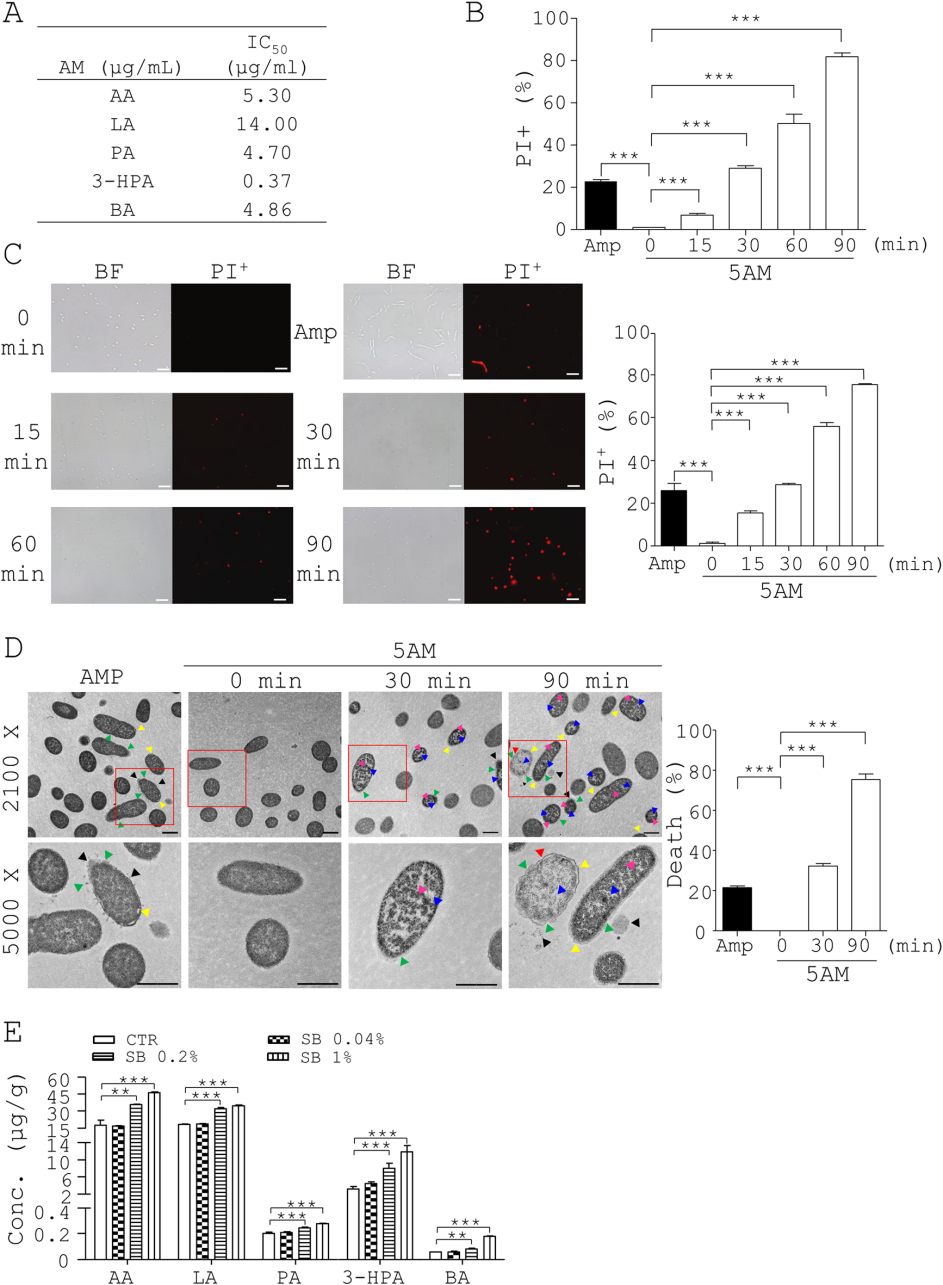
Anti-pathogenic mechanism of 5 antimicrobial metabolites produced by *L. johnsonii*. (**A**) The IC_50_ of each antimicrobial metabolite for *V. parahaemolyticus* (VP) was determined. (**B**–**C**) *V. parahaemolyticus* (VP) (1 × 10^6^ CFU/mL) were grown in TSB containing vehicle (NC), 30 µg/ml ampicillin (Amp), or each antimicrobial metabolite at the dosage that equaled their quantity in the supernatant of *L. johnsonii* for 90 min, followed by PI staining. The bacteria were divided into 2 aliquots. One aliquot of the bacteria was analyzed using flow cytometry (**B**). The other aliquot was analyzed with fluorescent microscopy (left, **C**). The percentage (%) of PI-positive (dead) cells was determined (right, **C**). (**D**) *V. parahaemolyticus* (VP) was treated with 30 µg/ml ampicillin and a mixture of the 5 antimicrobial metabolites which equaled the composition of the metabolites in the supernatant of SB-treated *L. johnsonii*, for the indicated times. The bacteria, *V. parahaemolyticus* were analyzed using TEM. Their representative images are shown (left). Arrowheads in yellow, red, white, and blue indicate separation of the cytoplasmic and outer membranes, distorted outer membrane, empty cells, and membrane discontinuity, respectively. Their death (%) was quantified and re-plotted into histograms (right). Scale bar = 1 µm. (**E**) Shrimp (86 days old) were fed with a standard diet (CTR) and a diet containing SB at the indicated dosages for 4 weeks. Their gut digesta were subjected to LC–MS/MS analysis, followed by identification and quantification of 5 antimicrobial metabolites (µg/g of digesta) as shown in Fig. S6 and Table S7. Data from 3 repeats are presented as the mean ± SD. One-way ANOVA test was used for statistical analysis of differences between groups and *P* (*) < 0.05, *P* (**) < 0.01, and *P* (***) < 0.001 are considered statistically significant.

To further understand the mechanism by which *Lactobacillus* inhibited *Vibrio*, agar well diffusion assays were performed to measure the antagonism between the supernatants of *L. johnsonii* and *L. reuteri* and *Vibrio* species. As expected, two negative controls, MRS medium and water, failed to inhibit *V. parahaemolyticus* as characterized by lack of an inhibition zone (NC1 and NC2, VP, Fig. 4D). In contrast, a positive control (PC), 0.125 µg Chl, inhibited the growth of *V. parahaemolyticus* as shown by inhibition zones (PC, VP, Fig. 4D). Likewise, the cell-free supernatant of *L. johnsonii* (LJSN) and *L. reuteri* (LRSN) significantly inhibited *V. parahaemolyticus* as shown by inhibition zones (LJSN and LRSN, VP, Fig. 4D). The supernatant of *L. johnsonii* had a slightly more potent inhibition of *V. parahaemolyticus* than that of *L. reuteri* and the positive control, 0.125 µg Chl (LJSN, LRSN, and PC, VP, Fig. 4D). In parallel, we performed antagonistic tests between the supernatant of two *Lactobacillus* species and *Vibrio* spp. using agar well diffusion assays. The supernatant of *L. johnsonii* had a slightly more potent inhibition of *Vibrio* spp. than that of *L. reuteri* and the positive control, 0.125 µg Chl (LJSN, LRSN, and PC, VS, Fig. 4D). Furthermore, we tried to identify the antibacterial metabolites present in the supernatant of *L. johnsonii* using LC–MS/MS. Consequently, five metabolites of *L. johnsonii*, LA, AA, PA, 3-HPA, and BA, were identified from the supernatant of *L. johnsonii* (CTR, Fig. 4E) compared to their standards (Fig. S5). Furthermore, LC–MS/MS data revealed that SB significantly up-regulated the above metabolites in the supernatant of *L. johnsonii* at the indicated time (Fig. 4E and Table S6) as well as in the gut microbiota (Fig. 5E and Table S7). We also evaluated the antimicrobial effects of the 5 metabolites on *V. parahaemolyticus* using agar well diffusion assays. No inhibition of *V. parahaemolyticus* growth was observed in a disc containing MRS medium and distilled water but inhibition was observed for Chl (NC1 and NC2 vs. PC, Fig. 4F), whilst the supernatant of *L. johnsonii* inhibited *V. parahaemolyticus* growth (LJSN, Fig. 4F) to a lesser degree than that of *L. johnsonii* with SB treatment (LJSN + SB, Fig. 4F). We also evaluated the antimicrobial potency of the 5 metabolites, in combination, at the dosage that equaled their quantity in the supernatant of *L. johnsonii*. A mixture of the 5 metabolites showed significant inhibition of *V. parahaemolyticus* growth (5AM, Fig. 4F). In addition, we tested the antimicrobial potency of a mixture of the 5 metabolites at the dosage that equaled their quantity in the *L. johnsonii* supernatant with SB treatment for 7 h. This mixture showed significant inhibition of *V. parahaemolyticus* growth (5AM + SB, Fig. 4F). Obviously, a mixture of the 5 metabolites (5AM + SB) and supernatant of SB-treated *L. johnsonii* (LJSN + SB) corresponding to their amount in SB treatment groups, had superior inhibition of *V. parahaemolyticus* compared to their amount in control groups (5AM and LJSN) (Fig. 4F). Overall, SB antagonized growth of *V. parahaemolyticus* through up-regulation of the antimicrobial metabolites from *L. johnsonii*.

### Antimicrobial metabolites of *L. johnsonii* suppress* V. parahaemolyticus* via membrane destruction

To investigate the antimicrobial mode of action of these metabolites produced by *Lactobacillus*, we assessed the bactericidal activities of individual metabolites toward *V. parahaemolyticus*. The IC_50_ values of the 5 compounds against *V. parahaemolyticus* in ascending order were: 3-HPA (0.37 µg/mL) < PA (4.70 µg/mL) < BA (4.86 µg/mL) < AA (5.30 µg/mL) < LA (14 µg/mL) (Fig. 5A). Next, we investigated cell death of *V. parahaemolyticus* using a flow cytometer and a microscope. Flow cytometry data showed that no cell death of *V. parahaemolyticus* before treatment with a mixture of the 5 metabolites was shown by propidium iodide (PI) staining (0 min, Fig. 5B). However, Amp induced death in *V. parahaemolyticus* (Amp, Fig. 5B). Consistently, a mixture of the 5 metabolites at the dosage that equaled their quantity in the supernatant of *L. johnsonii* caused a higher death of *V. parahaemolyticus* over time (VP, Fig. 5B). The above data was confirmed by fluorescent microscopy (5AM, Fig. 5C). Transmission electron microscopy (TEM) showed that, in the absence of the mixture of the 5 metabolites, the control bacteria had intact membranes and regular cytoplasm (0 min, Fig. 5D). In marked contrast, 30 min treatment with the mixture increased the percentage of damaged/dead cells in *V. parahaemolyticus* to 32.33% (30 min, Fig. 5D). Likewise, this percentage increased to 75.33% after 90 min treatment (90 min, Fig. 5D). We also confirmed the presence of the 5 metabolites in the digesta of control and SB-fed shrimp (Table S7 and Fig. 5E). The LC–MS/MS data showed that the amount of the 5 metabolites per gram in control shrimp guts in descending order was: LA (18.06 µg) > AA (17.13 µg) > 3-HPA (3.14 µg) > PA (0.20 µg) > BA (0.06 µg) (CTR, Fig. 5E). In contrast, SB dose-dependently increased the amount of these 5 metabolites. The amount of the 5 metabolites per gram of 1% SB-fed shrimp digesta in descending order was: AA (46.29 µg) > LA (34.53 µg) > 3-HPA (11.85 µg) > PA (0.28 µg) > BA (0.18 µg) (SB, Fig. 5E). Overall, SB up-modulated the level of 3-HPA, AA, and BA to a greater extent than LA and PA in shrimp guts. Overall, SB significantly escalated the level of these metabolites in shrimp.

### Identification of active compounds of SB with an ability to promote growth of *Lactobacillus*

**Figure 6:**
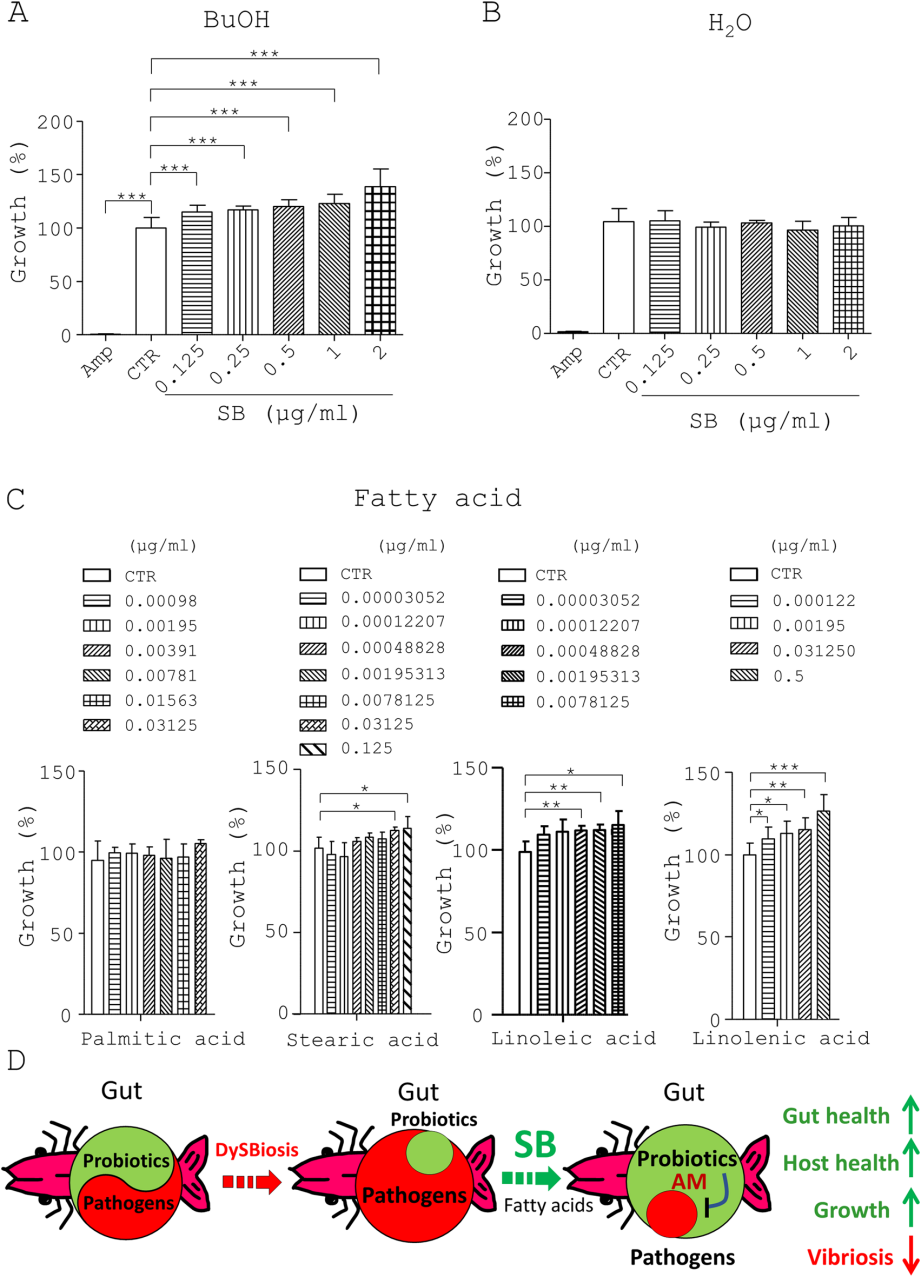
Identification of active compounds of SB with an ability to boost *Lactobacillus* growth. (**A**) The crude extract (CE) of SB was fractionated into butanol (BuOH) and water (H_2_O) fractions based on the bioactivity-directed fractionation and isolation procedure (Fig. S7). *L. johnsonii* (LJ) was cultured in MRS medium (CTR) and the medium containing ampicillin (Amp, 30 µg/ml) and the BuOH fraction at 0.125, 0.25, 0.5,1 and 2 µg/ml at 37 °C under anaerobic conditions for 10 h. The growth rate (%) of bacteria was obtained from the ratio of the OD_600_ of the treatment group to that of the control group multiplied by 100%. (**B**) The same procedure as (**A**) was conducted except that MRS medium (CTR) and the medium containing Amp, H_2_O fraction at 0.125, 0.25, 0.5, 1 and 2 µg/ml was used. (**C**) The same procedure as (**A**) was conducted except that palmitic acid, stearic acid, linoleic acid and linolenic acid at the indicated dosages were used. (**D**) In eubiosis, probiotics and pathogens are balanced in gut microbiota of healthy shrimp. In dysbiosis, probiotics are outcompeted by pathogens in shrimp gut and, in turn, this imbalance of microbiota causes host diseases. SB can promote the growth of probiotics and the production of their antimicrobial metabolites, leading to decreased pathogens. As a result, SB can enhance gut health, host health, and growth performance as well as rectify vibriosis and other disease in shrimp.

Finally, we tried to identify the active compounds from SB based on *L. johnsonii* growth. Using a bioactivity-directed fractionation and isolation strategy, we first obtained the crude extract (CE) of SB and then partitioned the CE into water and butanol (BuOH) fractions (Fig. S7A). The CE, BuOH fraction, and water fraction were incubated with *L. johnsonii* to assess their bioactivities. As a result, the CE and BuOH fraction but not water fraction promoted the growth of *L. johnsonii* (Fig. 6A and B and Table 4). Next, we purified 4 compounds from the BuOH fraction of SB, palmitic acid, linoleic acid, linolenic acid, and stearic acid, which were characterized using GC-GC/MS (Fig. S7B and Table 5). Meanwhile, we evaluated the MAC values of these 4 compounds based on *L. johnsonii* growth We found that linolenic acid had higher growth-promoting activity toward *L. johnsonii* than linoleic acid and stearic acid (Fig. 6C and Table 5). In contrast, palmitic acid showed no growth promotion activity toward *L. johnsonii* (Fig. 6C and Table 5)*.* Of note, the overall data demonstrated that SB promoted the growth performance and reduced vibriosis in shrimp via regulation of gut microbiota, *i.e.*, increased probiotics and reduced pathogens (Fig. 6D).

**Table 4:** Minimal activatory concentration for *L. johnsonii* on Shrimp Best and its different fractions.

Shrimp-Best (SB)	MAC_20_ (μg/ml)	Fold
CE	1.36	1
H_2_O	–	–
BuOH	0.6	2.3

**Table 5:** The composition of the active compounds in the crude extract (CE) and butanol fraction (BuOH) partitioned from Shrimp Best, and their minimal activatory concentration for *L. johnsonii*.

Proportion (%)	CE (%)	BuOH (%)	Fold (BuOH/CE)	MAC_20_ (μg/ml)
C16:0 (Palmitic acid)	0.49	1.07	2.18	–
C18:0 (Stearic acid)	0.03	0.07	2.01	0.24
C18:2 (Linoleic acid)	0.10	0.31	2.93	0.02
C18:3 (Linolenic acid)	0.29	1.00	3.47	0.24

In conclusion, SB was able to augment the growth performance and host defense to *Vibrio* in whiteleg shrimp. This augmentation arose from SB-mediated regulation of gut microbiota, *i.e.*, the promotion of probiotics and their antimicrobial metabolites, which counteracted the growth of pathogens. Accordingly, three active ingredients of SB were characterized for increased probiotic growth.

## Discussion

Shrimp aquaculture is facing multifaceted challenges. One major threat to aquatic animals is infections bacteria, virus, and fungus infection. Even though antibiotics are commonly used to prevent and treat bacterial infections, *Vibrio,* a causative pathogen of early mortality syndrome and white feces syndrome, is still a threat that causes considerable shrimp loss. Phytogenics are emerging as alternatives to antibiotics, although not many have been proved effective against *Vibrio* and other infections. In this study, we demonstrated that SB, composed of Spanish needles and bitter melon, ameliorated host health, growth performance, and defense against *Vibrio* infection in shrimp. The effective dosage of SB was as low as 0.2% for growth and host defense in shrimp (Figs. 1 and 2), proving its feasibility in aquaculture. More importantly, the efficacy of SB was confirmed in a field trial (Table 1). SB could have different functions in host and gut pathogens of shrimp due to its multiple phytochemicals. For example, the Asteraceae, *B. pilosa*, a component of SB, has been reported to possess over 40 distinct bioactivities^32^. This study showed that SB and its active compounds could modulate intestinal probiotic bacteria and pathogens, leading to the maintenance of eubiosis in shrimp gut microbiota (Fig. 6D). This SB-implicated mechanism is further supported by publications stating that oral administration of *Lactobacillus* improved host health, growth performance and FCR in whiteleg shrimp^33^. Accordingly, this administration reduced the mortality of shrimp when challenged with *V. parahaemolyticus*^33^. Of note, SB had the edge over probiotic supplementation in up-regulation of endogenous probiotics (but not exogenous probiotics) in shrimp gut, and had more richness of gut probiotics than exogenous probiotic supplementation*.* Our research may thus constitute a novel phytogenic approach to boost host growth, health, and defense in shrimp.

Balance of gut microbiota is important for host health, disease reduction, growth, and reproduction^16^. However, multiple factors such as food, drugs, exogenous bacteria, host genetics, and stress, contribute to gut dysbiosis^34^. Thus, manipulation of gut microbiota from dysbiosis toward eubiosis is a promising strategy to prevent and treat diseases in hosts^35^. It is widely known that oral use of probiotics and prebiotics is beneficial for disease decrease, health maintenance, growth, and reproduction, involving increased probiotics in the host gut. Of note, this work showed that SB at 0.5 μg/mL and more significantly increased growth of *L. johnsonii* (Fig. 4A). Conversely, SB at 200 μg/mL and more significantly inhibited growth of *V. parahaemolyticus* (Fig. 4B). These data suggest that SB reduced *V. parahaemolyticus* via indirect antagonism between both bacteria (Fig. 4C and D). Of note, the dosage of SB used for *L. johnsonii* but not *V. parahaemolyticus* was easily achievable in the shrimp guts (Fig. 5E) because the content of 0.2% SB for growth roughly equaled 400 μg per 3 g shrimp (~ 133 μg/mL). Furthermore, linoleic acid, linolenic acid and stearic acid were identified as active compounds for the growth of probiotics (Figs. 6C and S7). The overall data suggest that SB play an eubiotic role in regulation of gut microbiota in shrimp. In parallel, we found that probiotics (*e.g.*, *Lactobacillus*) and their antimicrobial metabolites (*e.g.*, LA, AA, PA, BA and3-HPA) suppressed pathogens (*e.g.*, *Vibrio*) (Figs. 3 and 4). Interestingly, SB dose-dependently up-regulated the growth of probiotics (Fig. 3) and the production of these short-chain fatty acids (SCFA) (Figs. 4E and 5E and Tables S6 and S7). The above data supported this interbacterial antagonism between *Lactobacillus* and *Vibrio* (Figs. 4 and 5). In fact, the SB-mediated regulation of gut microbiota in shrimp was similar to regulation of gut microbiota in chickens by *B. pilosa*^20^. However, we cannot exclude the possibility that SB decreased pathogens directly via its antimicrobial phytochemicals. We also noted that SB increased the number of *Lactobacillus* (Fig. 4A) to a lesser degree than the production of metabolites in its supernatant (Table S6). However, whether this increase was ascribed to the up-regulation of the *Lactobacillus* number and/or the overproduction of antimicrobial metabolites needs to be verified.

Over 1340 plants and their compounds have been reported to possess antimicrobial activity, including *B. pilosa* and *M. charantia*^36^. However, little is known about their antibacterial mechanism in hosts. For the first time, we revealed that SB, a phytogenic that contains extracts of both plants, controlled pathogens via increasing probiotics and, subsequently, inhibiting of pathogens (Fig. 6D). Astonishingly, this control mechanism involved the antagonism of SB-mediated probiotics toward pathogens rather than direct killing of pathogens by SB. Compared to other antimicrobial phytogenics, SB did suppress gut pathogens through its ability to augment probiotic growth and production of probiotic SCFA. Nevertheless, the limitations and challenges faced in the development of antimicrobial phytogenics include difficulty in identifying anti-bacterial compounds from plants, uncertain mode of action, ineffective bioassays, and so on^37^. We combined *L. johnsonii* culture and phytochemistry to identify the active compounds from SB by using a bioactivity-directed fractionation and isolation approach. As a result, we successfully identified 4 fatty acids with 16 to 18 carbons from SB. These fatty acids were reported to be present in *B. pilosa*^24^. The data showed that the fatty acids with 18 carbons increased *Lactobacillus* growth (Fig. 6C). Surprisingly, palmitic acid had no detectable activity toward *Lactobacillus.* Although we could not rule out the existence of other active compounds in SB, we clearly characterized some compounds as active compounds. These active compounds can be used to explain the function and mechanism of SB and serve as index compounds for the quality control of SB. Like antibiotics, most plants and compounds have been shown to directly kill and/or suppress pathogens^24^, thus raising the possibility of antimicrobial resistance. However, SB did not directly kill pathogens but directed the expansion of probiotics and, therefore, outcompeted pathogens, leading to control of pathogens in the guts of shrimp. Apparently, one extra advantage of SB is the unlikeliness of development of drug resistance in pathogens in response to SB. Thus, SB can be considered to have a unique function and mechanism for the control of gut microbiota. Moreover, SB has no antibiotic residue and antibiotic resistance, which is particularly appropriate for antibiotic-free aquaculture.

### Supplementary Information

Supplementary Information.

## Data Availability

The raw data supporting the conclusions of this manuscript are included in the supplementary information.
