# Supplementary material for: Functional and mechanistic studies of a phytogenic formulation, Shrimp Best, in growth performance and vibriosis in whiteleg shrimp
Source: Sci Rep. 2024 May 21;14:11584. doi: 10.1038/s41598-024-62436-x (PMC11109214; doi:10.1038/s41598-024-62436-x)
Supplement: Supplementary file 1 — Supplementary Information. [file 41598_2024_62436_MOESM1_ESM.pdf]

Supplementary information

Functional and mechanistic studies of a phytogenic formulation, Shrimp Best, in growth performance and vibriosis in whiteleg shrimp

Yi-San Lee[1,2#], Khotibul Umam[1,3,4,5#], Tien-Fen Kuo[1], Yu-Liang Yang[1], Ching-Shan Feng[1], and Wen-Chin Yang[1,3,4,6 *]

A. SUPPLEMENTARY FIGURES

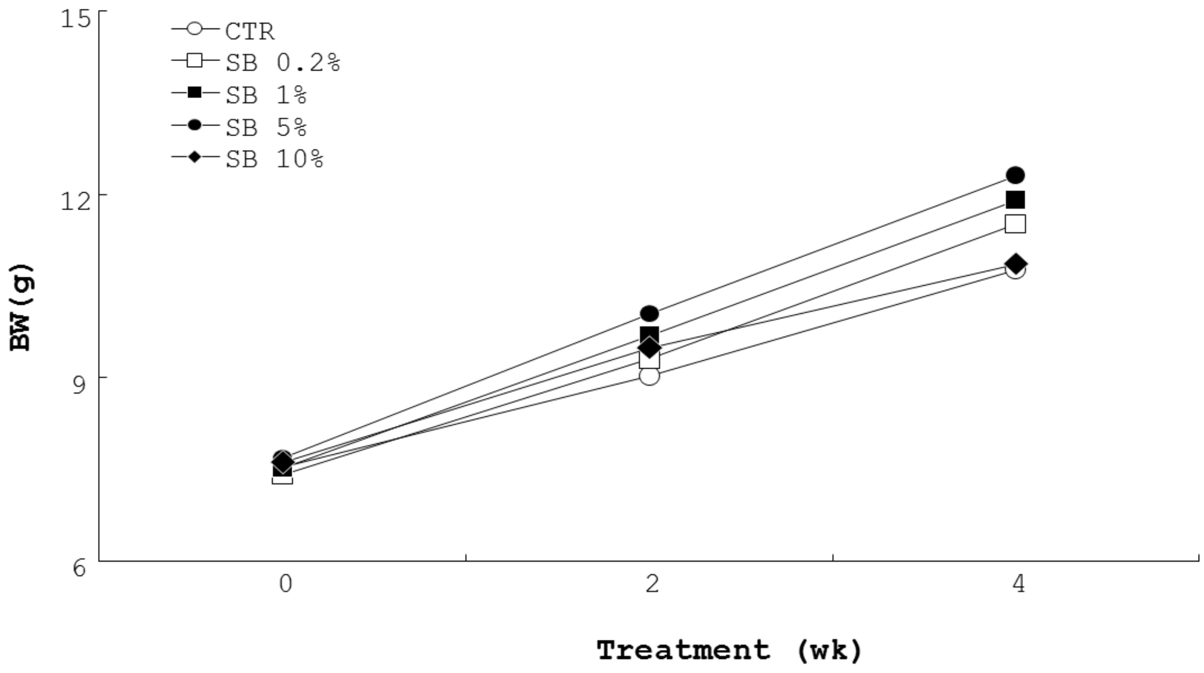

Figure S1. Toxicity of SB in shrimp. Five groups of 110-day-old shrimp, 30 animals per group, were fed with a standard diet (CTR) or a diet containing SB at 0.2%, 1%, 5% and 10% for 4 weeks. Their body weight (BW) was measured for toxicity assessment.

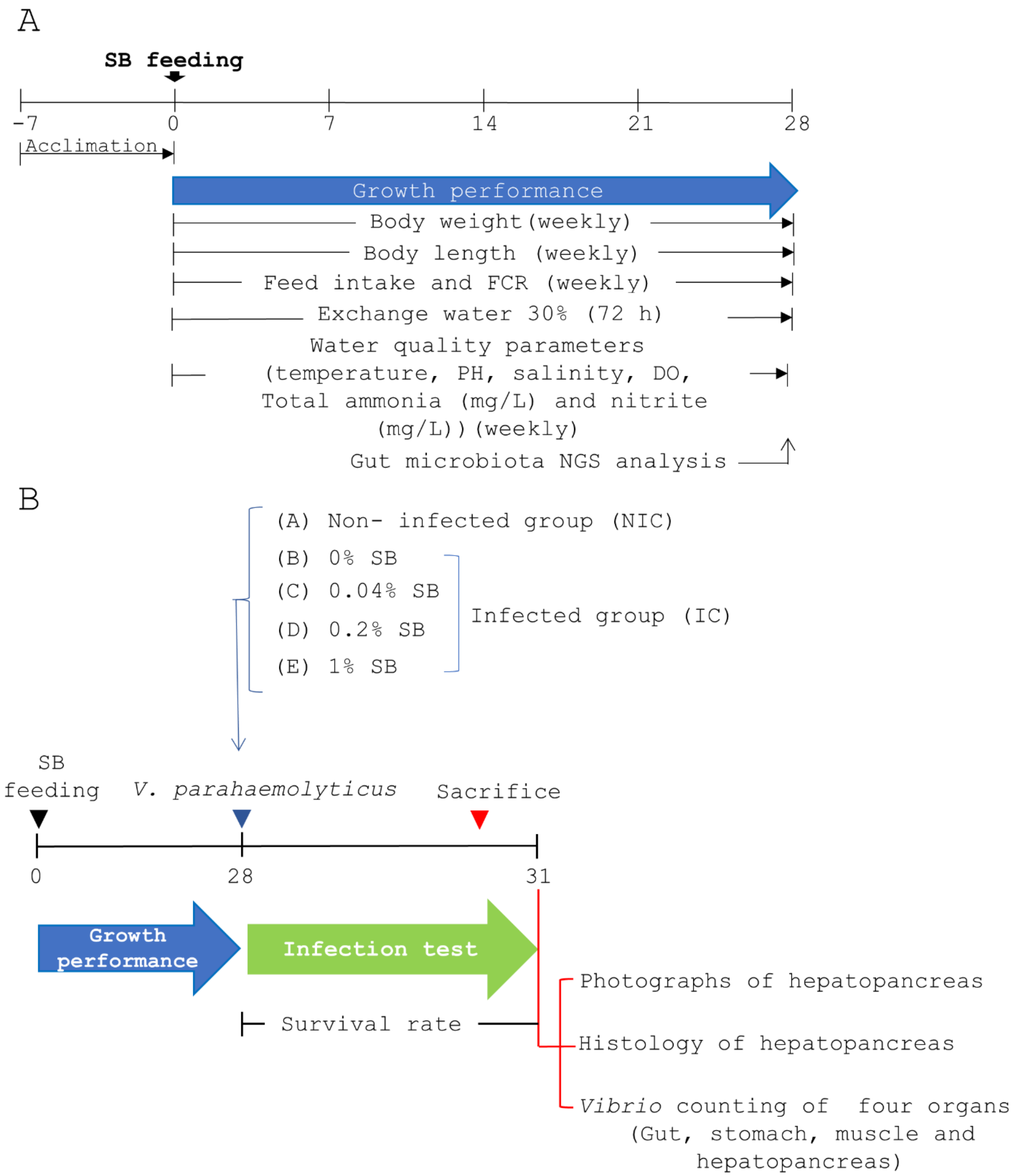

Figure S2. Experimental design use for growth performance, analysis of gut microbiota, and *Vibrio* infection in whiteleg shrimp. (A) A flow chart of the experimental design used in the laboratory study. (B) A flow chart of the experimental design used in the challenge study. Control shrimp (NIC) were not infected with *V. parahaemolyticus*. The infected groups (IC) received an intramuscular injection of *V. parahaemolyticus* (5x10^4 CFU/g BW).

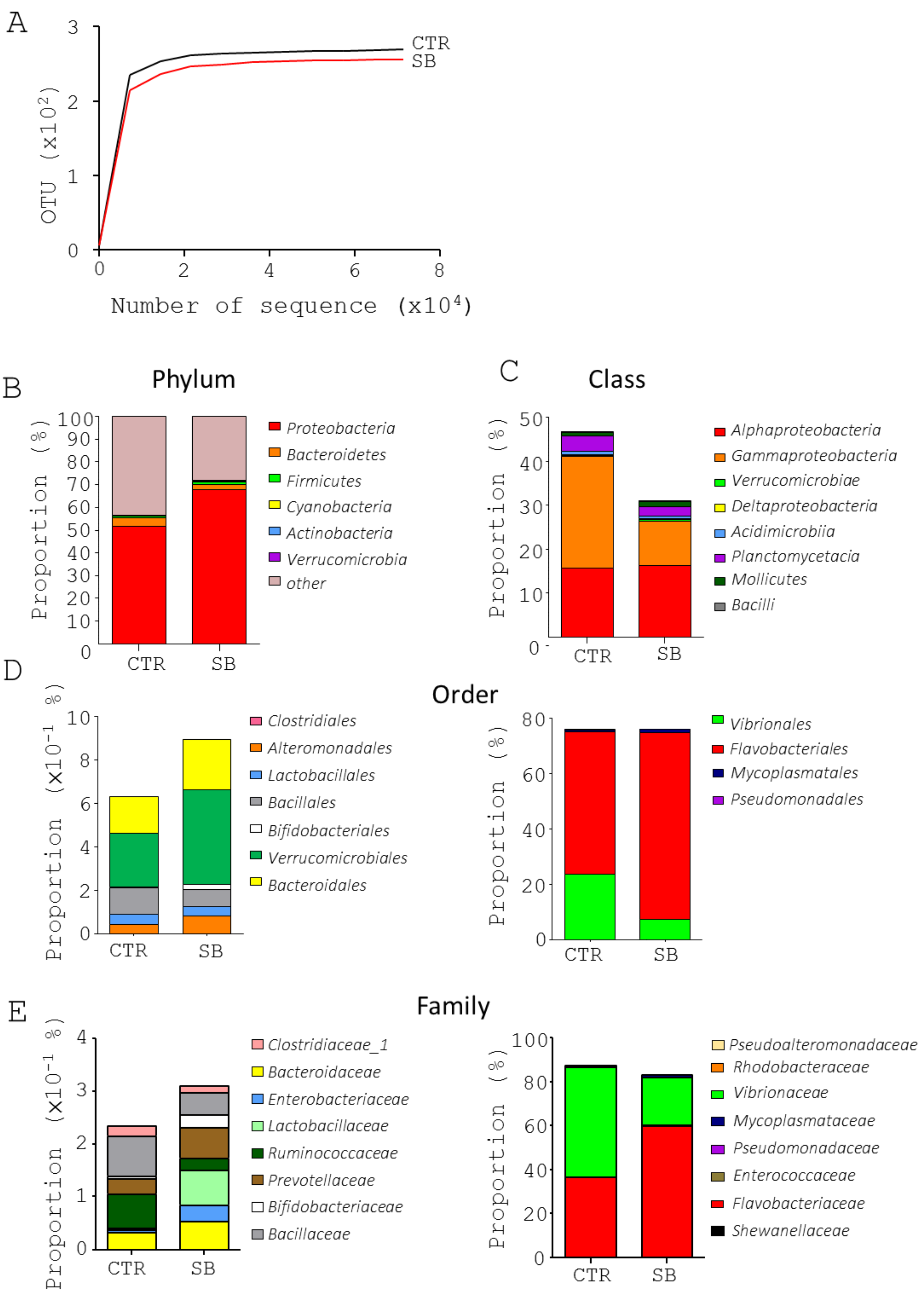
A
OTU (x10²)
CTR
SB
Number of sequence (x10⁴)
B
Phylum
Proportion (%)
Proteobacteria
Bacteroidetes
Firmicutes
Cyanobacteria
Actinobacteria
Verrucomicrobia
other
C
Class
Alphaproteobacteria
Gammaproteobacteria
Verrucomicrobiae
Deltaproteobacteria
Acidimicrobiia
Planctomycetacia
Mollicutes
Bacilli
D
Order
Proportion (x10⁻¹ %)
Clostridiales
Alteromonadales
Lactobacillales
Bacillales
Bifidobacteriales
Verrucomicrobiales
Bacteroidales
Vibrionales
Flavobacteriales
Mycoplasmatales
Pseudomonadales
E
Family
Clostridiaceae_1
Bacteroidaceae
Enterobacteriaceae
Lactobacillaceae
Ruminococcaceae
Prevotellaceae
Bifidobacteriaceae
Bacillaceae
Pseudoalteromonadaceae
Rhodobacteraceae
Vibrionaceae
Mycoplasmataceae
Pseudomonadaceae
Enterococcaceae
Flavobacteriaceae
Shewanellaceae

Figure S3. Experimental design and analysis of 16S rRNA sequencing analysis of gut microbiota in shrimp. (A) Rarefaction curves of bacterial OTUs in the fecal bacteria of control shrimp (CTR) and shrimp fed with 1% SB (Fig. 1) using the 16S rRNA NGS analysis. (B-E) The 16S rRNA NGS analysis indicated the relative abundance of gut bacteria at the level of phylum (B), class (C), order (D) and family (E).

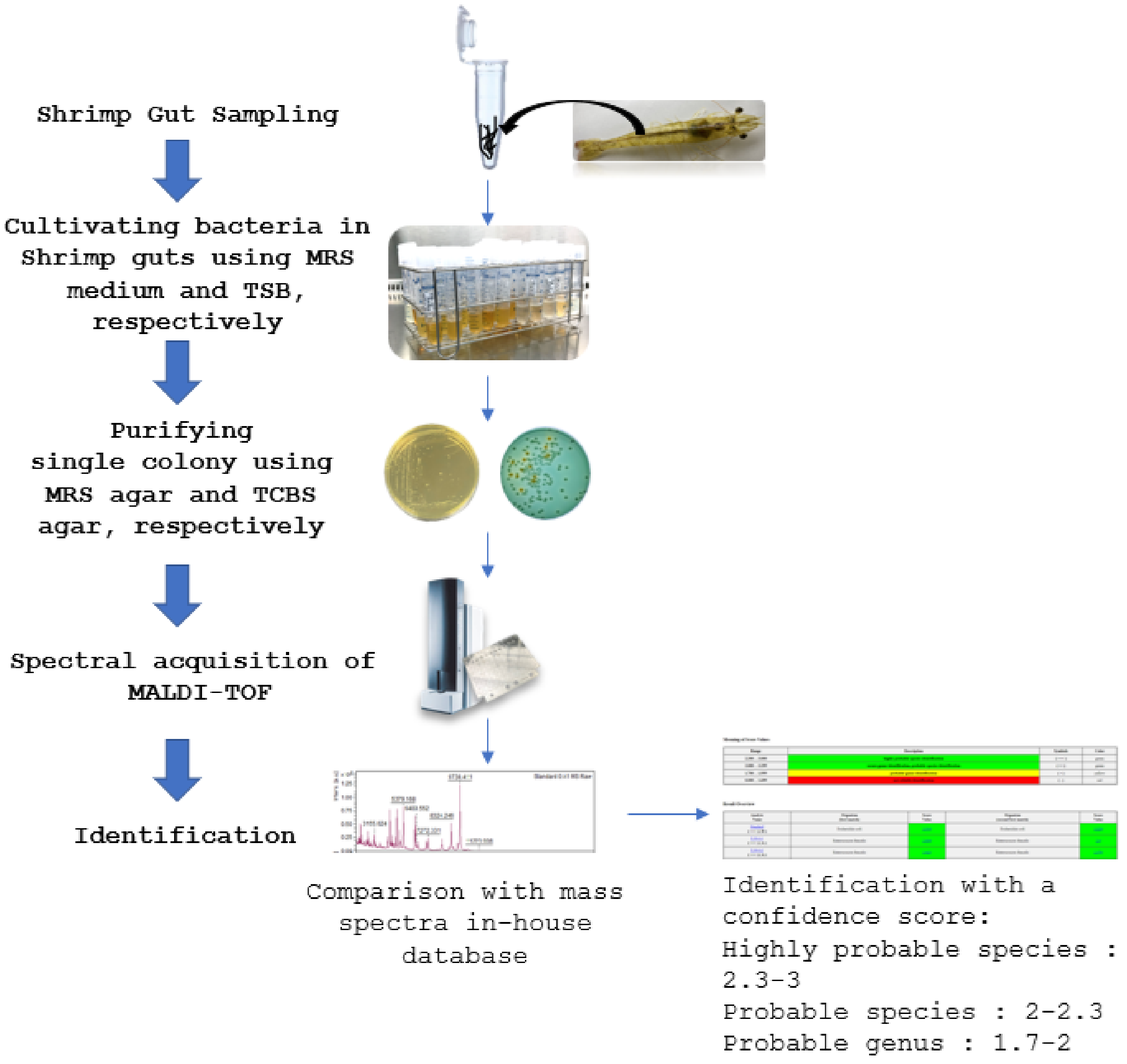

Figure S4. Intestinal bacteria isolation, screening, and identification of probiotics and pathogens from shrimp gut. The procedure of intestinal bacteria collection, cultivation, screening single colony, MALDI-TOF MS analysis and identification using Biotype 3.1 against an in-house database. A mixture of three shrimp guts was collected and screened for 16-48 single strains of probiotics and pathogens. Then, the single strains were identified by MALDI-TOF MS analysis.

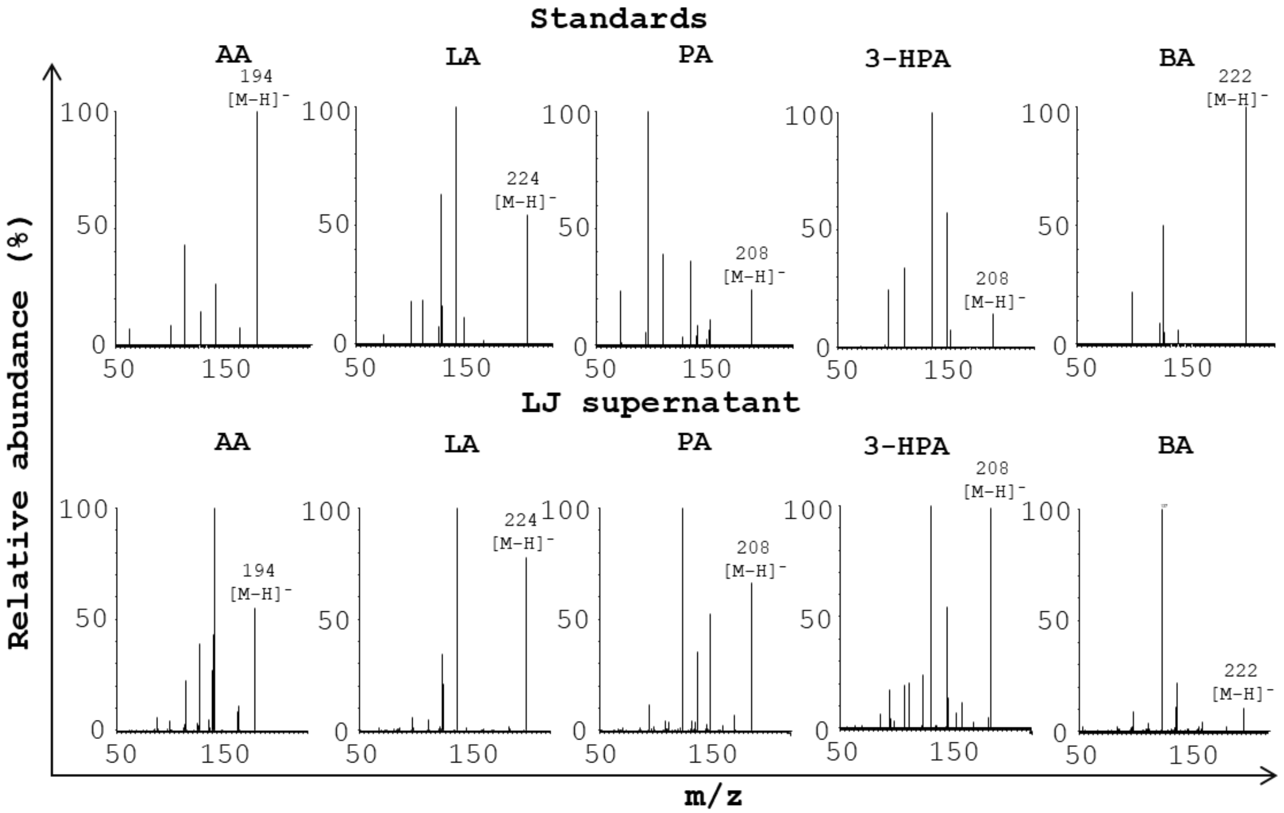

Figure S5. Identification of five antimicrobial metabolites (5AM) produced by *L. johnsonii*. The 5AM [acetic acid (AA), butyric acid (BA), lactic acid (LA), propionic acid (PA), and 3-hydroxypropionic acid (3-HPA)] in the supernatant of *L. johnsonii* grown in MRS broth (bottom) as well as the standards (top), were subjected to LC-MS/MS. Representative MS/MS profiles of the 5 ion signals corresponding to the 5AM are indicated. The quantification of antimicrobial metabolites (AM) in each group was obtained based on the area under the curve of LC histograms (Fig. 4E).

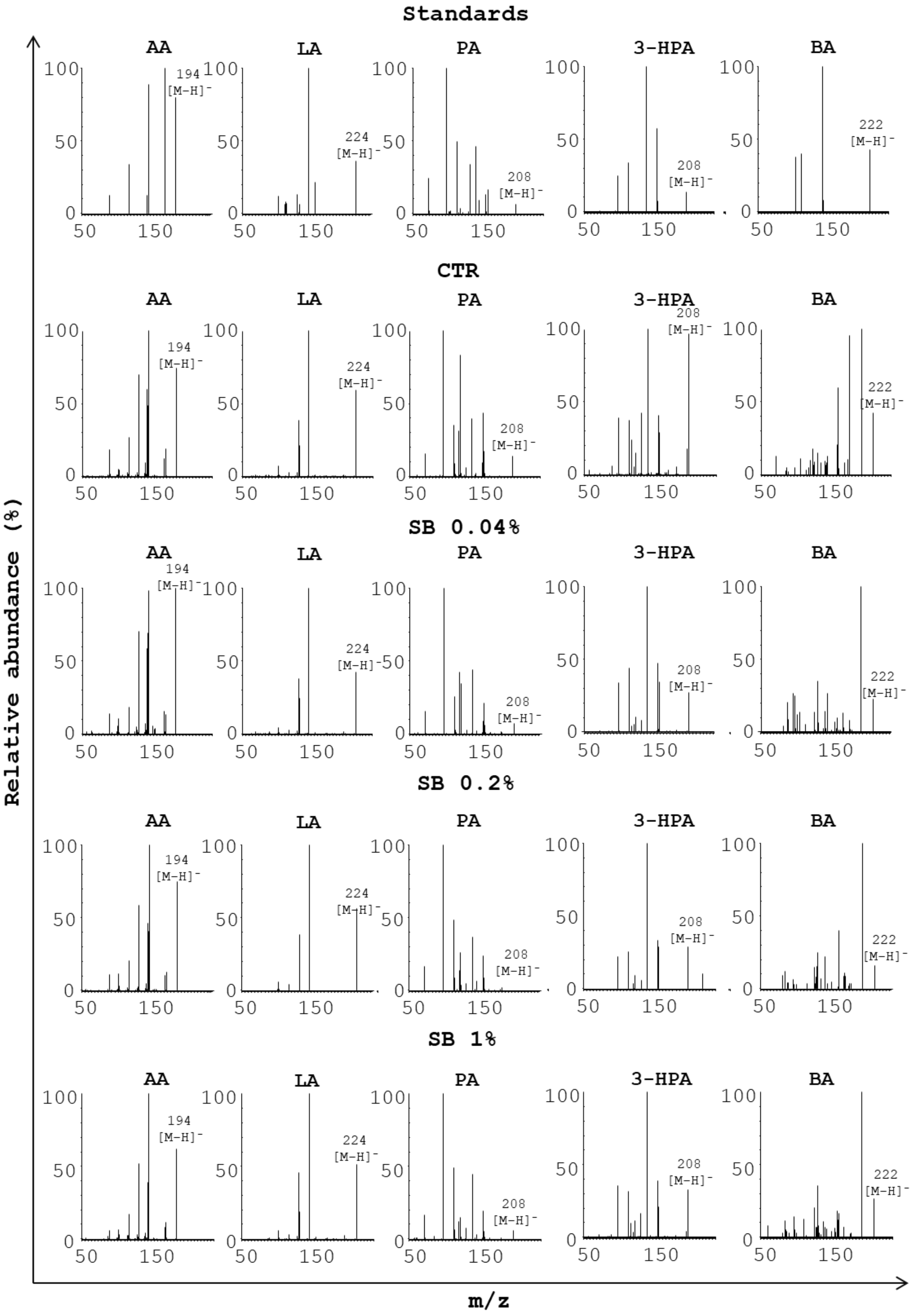
Standards
CTR
SB 0.04%
SB 0.2%
SB 1%
AA
LA
PA
3-HPA
BA
194 [M-H]⁻
224 [M-H]⁻
208 [M-H]⁻
222 [M-H]⁻
0
50
100
150
Relative abundance (%)
m/z

Figure S6. Identification of five antimicrobial metabolites (5AM) in the shrimp guts. Eighty-six-day-old shrimp were fed with a standard diet (CTR) and a diet containing SB at the three indicated dosages for 4 weeks. Their gut digesta were subjected to LC-MS/MS analysis, followed by quantification of 5AM [acetic acid (AA), butyric acid (BA), lactic acid (LA), propionic acid (PA), and 3-hydroxypropionic acid (3-HPA)] (μg/g of guts). Representative MS1 profiles of the 5 ion signals corresponding to the 5AM standards are indicated (1st row). MS/MS profiles of the 5AM present in control shrimp gut (CTR, 2nd row) and three SB-fed shrimp gut (3rd to 5th rows) are shown. The quantification of 5AM in each group was obtained based on the area under the curve of LC histograms (Fig. 5E).

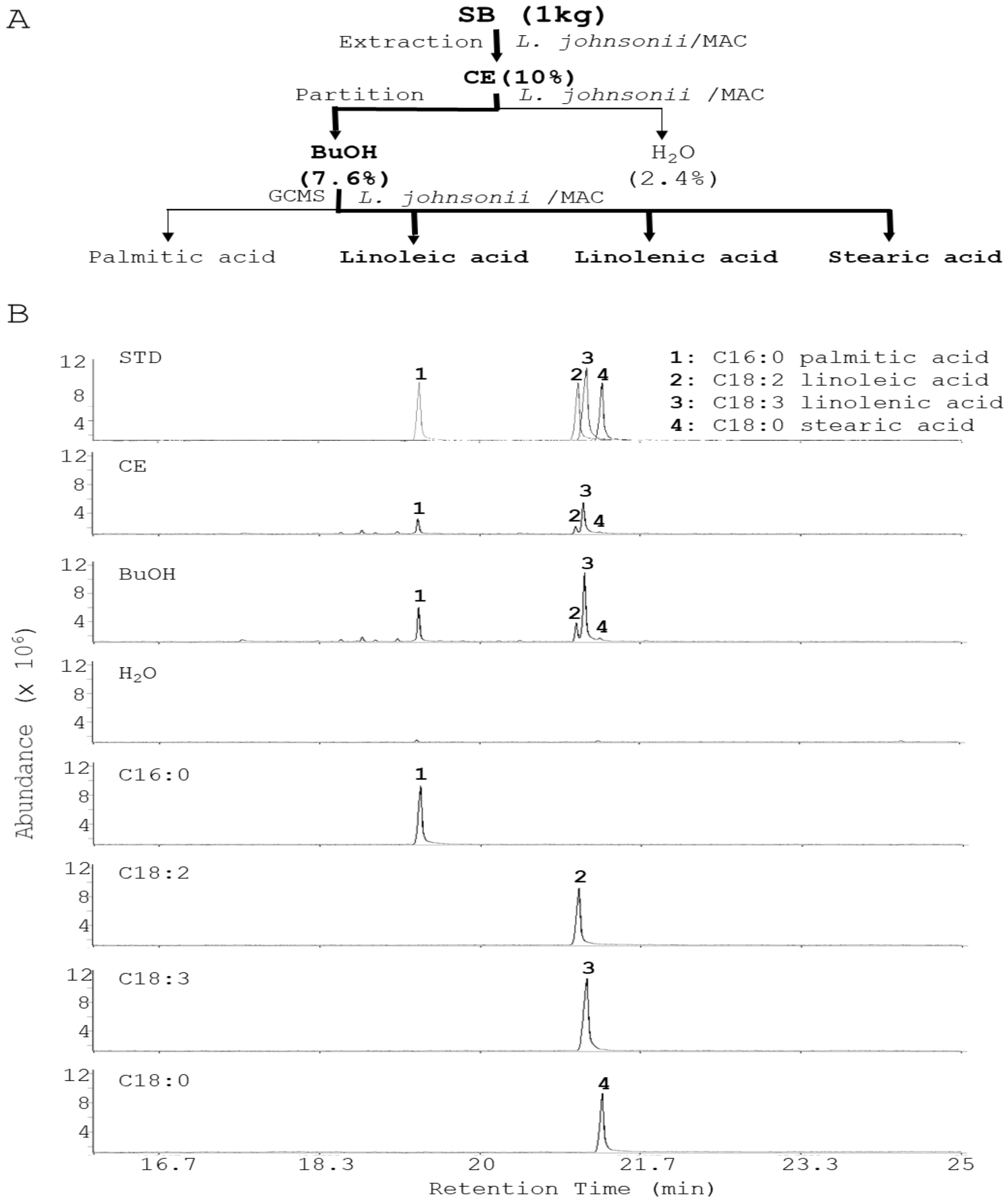

Figure S7. Identification and quantification of active compounds of SB. (A) We partitioned the crude extract (CE) of SB into butanol (BuOH) and water (H_2O) fractions. Using a bioactivity-directed fraction and isolation strategy, we purified and identified four active compounds, including palmitic acid (**1**), linoleic acid (**2**), linolenic acid (**3**), and stearic acid (**4**), which were able to promote *L. johnsonii* growth, from the SB extract. (B) GC-GC/MS analysis was used to characterize the four fatty acids in standards (STD), CE, BuOH fraction, H_2O fraction, and pure compounds of SB.

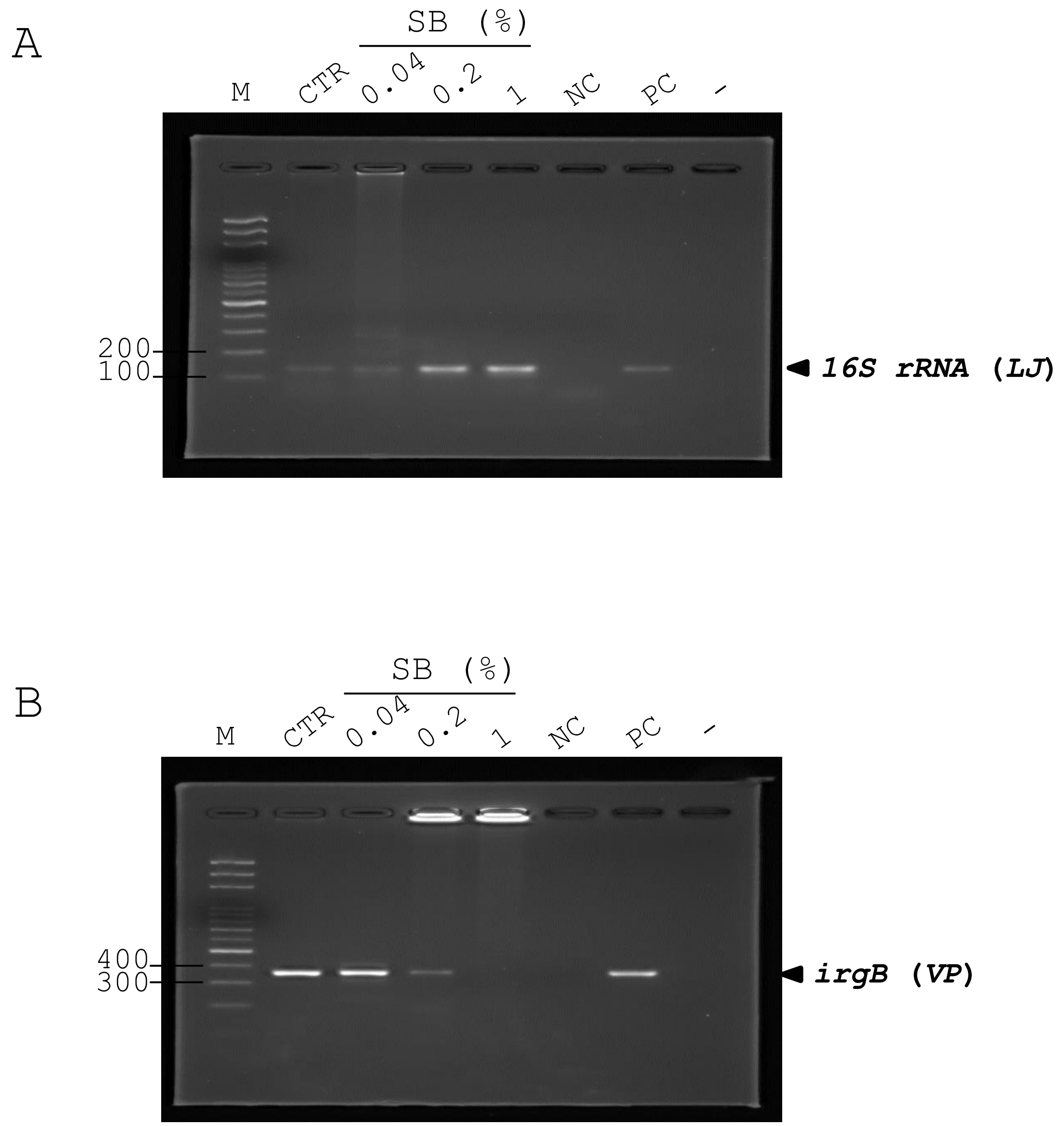

Figure S8. Full-length gel of Fig. 3E and 3F. DNA marker (M) and PCR amplicon of gut bacterial DNA of shrimp fed with SB (0.04%, 0.2%, and 1%), water (NC), and DNA (PC) of *L. johnsonii* (A) or *Vibrio parahaemolyticus* (B). The rightmost lane (-) is empty.

B. SUPPLEMENTARY TABLES

Table S1. Growth performance of shrimp in a 4-week laboratory study.

	CTR	SB 0.04%	SB 0.2%	SB 1%
Initial weight (g)	3.26 ± 0.29	3.27 ± 0.32	3.29 ± 0.34	3.31 ± 0.28
Initial length (mm)	65.75 ± 2.67	65.85 ± 2.21	65.68 ± 2.57	66.11 ± 2.53
Final weight (g)	4.53 ± 0.03	4.63 ± 0.12^{a}	5.09 ± 0.04^{a,b}	5.41 ± 0.03^{a,b,c}
Final length (mm)	71.04 ± 3.78	71.54 ± 3.72^{a}	75.30 ± 2.59^{a,b}	76.87 ± 3.71^{a,b}
Body weight gain (g)	1.27 ± 0.04	1.36 ± 0.12^{a}	1.81 ± 0.03^{a,b}	2.10 ± 0.03^{a,b,c}
Specific growth rate (%/day)	4.53 ± 0.15	4.87 ± 0.42^{a}	6.46 ± 0.10^{a,b}	7.49 ± 0.12^{a,b,c}
Feed intake (g)	2.33 ± 0.05	2.47 ± 0.10^{a}	2.83 ± 0.01^{a,b}	3.11 ± 0.02^{a,b,c}
Feed conversion ratio (FCR)	1.89 ± 0.15	1.79 ± 0.06^{a}	1.57 ± 0.03^{a,b}	1.48 ± 0.01^{a,b,c}

Results are presented as mean ± standard deviation. One-way ANOVA was used to compare difference of mean among CTR, SB 0.04%, SB 0.2% and SB 1%. The letters a, b, and c indicate statistical significance ($P < 0.05$) between the CTR and the SB-treated groups, SB 0.04%, SB 0.2%, and SB 1%, respectively.

Table S2. Water quality parameters in a 4-week laboratory study.

Parameters	CTR	SB 0.04%	SB 0.2%	SB 1%	Range*
Temperature (°C)	28.15 ± 0.99	28.55 ± 1.08	28.33± 1.11	28.11 ± 0.75	25 - 32
Salinity (‰)	15.17 ± 1.11	15.35 ± 1.05	15.96± 1.09	15.72 ± 0.71	10 - 25
pH	7.96 ± 0.31	8.22 ± 0.14	8.56 ± 0.46	8.02 ± 0.22	7.5 - 8.5
Dissolved Oxygen (mg/ml)	9.67 ± 1.93	9.67 ± 0.57	9.36 ± 1.00	9.54 ± 1.33	>4
Nitrite(mg/ml)	0.43 ± 0.07	0.66 ± 0.16	0.35 ± 0.16	0.55 ± 0.18	<1
Total ammonia (mg/ml)	0.29 ± 0.26	0.19 ± 0.13	0.12 ± 0.02	0.22 ± 0.12	<1

Results are presented as mean ± standard deviation. One-way ANOVA was used to compare the difference of mean among CTR, SB 0.04%, SB 0.2% and SB 1%. No statistical significance ($P < 0.05$) between the CTR and the SB-treated groups, SB 0.04%, SB 0.2%, and SB 1%, respectively, was observed. * The suitable range of water parameters for shrimp were checked with the publications[1, 2].

Table S3. Water quality parameters in a 135-day field trial.

Parameters	CTR	SB 0.2%	SB 1%	Range*
Temperature (°C)	29.80 ± 0.69	30.10 ± 0.61	30.57 ± 1.27	25 - 32
Salinity (‰)	20.28 ± 0.5	20.39 ± 0.39	20.04 ± 1.06	10 - 25
pH	7.92 ± 0.20	7.89 ± 0.69	8.39 ± 0.49	7.5 - 8.5
Dissolved oxygen (mg/ml)	9.32 ± 1.29	9.64 ± 0.94	9.77 ± 0.83	>4

Nitrite (mg/ml)	0.70 ± 0.19	0.80 ± 0.27	0.53 ± 0.19	<1
Total ammonia (mg/ml)	0.49 ± 0.29	0.49 ± 0.33	0.45 ± 0.25	<1

Results are presented as mean ± standard deviation. One-way ANOVA was used to compare the difference of mean among CTR, SB 0.04%, SB 0.2% and SB 1%. No statistical significance ($P < 0.05$) between the CTR and the SB-treated groups, SB 0.04%, SB 0.2%, and SB 1%, respectively, was observed. *The suitable range of water parameters for shrimp were checked with the publications[1, 2].

Table S4. The number of sequences, operational taxonomic units (OTUs), and diversity indices in shrimp gut digesta.

Sample	Number of sequences	Observed OTUs	Shannon diversity	Simpson diversity	ACE	Chao1 richness	Coverage
CTR	38170	332	3.443	0.737	371.016	381.763	0.998
SB 1%	38170	311	2.738	0.564	341.331	351.182	0.999

Table S5. The composition of gut microbiota at the phylum, class, order, family, and genus level in shrimp fed with SB for 28 days during a 4-week laboratory study.

Taxonomy	Name	Proportion in total sequences (%)	
		CTR	SB 1%
Phylum	*Proteobacteria*	51.708	67.692
	Bacteroidetes	3.591	2.166
	Firmicutes	0.788	1.205
	Cyanobacteria	0.168	0.361
	Actinobacteria	0.267	0.456
	Verrucomicrobia	0.024	0.008
Class	*Alphaproteobacteria*	15.672	16.317
	Gammaproteobacteria	25.440	10.051
	Verrucomicrobiae	0.267	0.456
	Deltaproteobacteria	0.189	0.068
	Acidimicrobiia	0.757	0.652
	Planctomycetacia	3.510	2.103
	Mollicutes	0.788	1.205
	Bacilli	0.168	0.123
Order	*Clostridiales*	0.002	0.001
	Alteromonadales	0.042	0.081
	Lactobacillales	0.047	0.045
	Bacillales	0.121	0.079
	Bifidobacteriales	0.005	0.024
	Verrucomicrobiales	0.246	0.435

	Bacteroidales	0.168	0.231
	Vibrionales	23.452	7.348
	Flavobacteriales	51.354	67.330
	Mycoplasmatales	0.778	1.205
	Pseudomonadales	0.144	0.149
Family	*Bacteroidaceae*	0.031	0.052
	Lactobacillaceae	0.005	0.031
	Enterobacteriaceae	0.003	0.066
	Ruminococcaceae	0.065	0.024
	Prevotellaceae	0.029	0.058
	Bifidobacteriaceae	0.005	0.024
	Bacillaceae	0.076	0.042
	Clostridiaceae_1	0.018	0.013
	Flavobacteriaceae	36.303	59.883
	Rhodobacteraceae	0.081	0.330
	Vibrionaceae	50.084	21.658
	Mycoplasmataceae	0.778	1.205
	Pseudomonadaceae	0.079	0.100
	Enterococcaceae	0.000	0.000
	Pseudoalteromonadaceae	0.000	0.000
	Shewanellaceae	0.000	0.000
Genus	*Lactobacillus*	0.005	0.031
	Megamonas	0.008	0.003
	Ruminococcus	0.000	0.003
	Megasphaera	0.000	0.011
	Alistipes	0.000	0.000
	Butyricicoccus	0.000	0.000
	Bifidobacterium	0.005	0.024
	Prevotella	0.050	0.115
	Collinsella	0.005	0.018
	Photobacterium	0.049	0.000
	Pseudoalteromonas	0.008	0.005
	Planctomicrobium	1.153	0.097
	Tenacibaculum	0.000	0.000
	Pirellula	0.018	0.084
	Corynebacterium_1	0.031	0.010
	Bythopirellula	0.045	0.045
	Rubripirellula	0.000	0.026
	Blastopirellula	0.186	0.220
	Vibrio	18.512	7.308

Table S6. Level of antimicrobial metabolites in the supernatant of *L. johnsonii in vitro*.

Metabolite (µg/ml)	CTR	SB
AA	7,760.00 ± 22.24	8,191.70 ± 216.98***
LA	2,278.27 ± 44.30	2,633.16 ± 223.75***
PA	5.90 ± 0.34	8.66 ± 2.23***
BA	12.68 ± 0.59	14.46 ± 1.20**
3-HPA	3,718.91 ± 185.99	4,466.83 ± 263.44**

The supernatant of L. johnsonii was grown in MRS medium containing 0.00025 µg/ml methanol (CTR) or SB at 2 µg/ml at the indicated times, then LC-ESI-MS was used for analysis. Data from 3 experiments are presented as mean ± standard deviation. One-way ANOVA: P (*) < 0.05, P (**) < 0.01, and P (***) < 0.001 were considered statistically significant.

Table S7. The contents of AM in the intestine of *L. vannamei* that were fed with standard diet and the diet containing SB for 28 days (µg/g) *in vivo*.

Metabolite (µg/g)	CTR	SB 0.04%	SB 0.2%	SB 1%
AA	17.13 ± 4.50	16.70 ± 0.20	35.61 ± 0.31[a, b]	46.29 ± 0.64[a, b, c]
LA	18.06 ± 0.33	22.46 ± 7.13	31.87 ± 1.11[a, b]	34.53 ± 0.87[a, b, c]
PA	0.20 ± 0.01	0.22 ± 0.02	0.25 ± 0.00[a, b]	0.28 ± 0.00[a, b, c]
BA	0.06 ± 0.00	0.07 ± 0.01	0.08 ± 0.01[a, b]	0.18 ± 0.00[a, b, c]
3HPA	3.14 ± 0.62	5.37 ± 1.18	7.98 ± 1.21[a, b]	11.85 ± 1.52[a, b, c]

Results are presented as mean ± standard deviation. One-way ANOVA was used to compare the difference of mean among CTR, SB 0.04%, SB 0.2% and SB 1%. The letters a, b, and c indicate statistical significance (*P* < 0.05) between the CTR and the SB-treated groups, SB 0.04%, SB 0.2%, and SB 1%, respectively.

References:

1. Apud, F.D. Recent developments in prawn pond culture. https://repository.seafdec.org.ph/bitstream/handle/10862/1483/aep01.pdf;jsessionid=206CBDC894AF01A44586A2D0D7741E54?sequence=1 (1989).

2. Margabandu, V. & Ramamurthy, D. Recent farming practices for culturing sustainable pacific White Shrimp, Peneaus vannamei. *Int. J. Sci. Res.* **4**, 9-12.(2015).
